# The genetic architecture of breast papillary lesions as a predictor of progression to carcinoma

**DOI:** 10.1038/s41523-020-0150-6

**Published:** 2020-03-12

**Authors:** Tanjina Kader, Kenneth Elder, Magnus Zethoven, Timothy Semple, Prue Hill, David L. Goode, Niko Thio, Dane Cheasley, Simone M. Rowley, David J. Byrne, Jia-Min Pang, Islam M. Miligy, Andrew R. Green, Emad A. Rakha, Stephen B. Fox, G. Bruce Mann, Ian G. Campbell, Kylie L. Gorringe

**Affiliations:** 1 Peter MacCallum Cancer Centre, Melbourne, VIC Australia; 2 The Sir Peter MacCallum Department of Oncology, University of Melbourne, Parkville, VIC Australia; 3 The Breast Service, The Royal Women’s Hospital, Fitzroy, VIC Australia; 4 Department of Anatomical Pathology, St Vincent’s Hospital, Fitzroy, VIC Australia; 5 Nottingham Breast Cancer Research Centre, Division of Cancer and Stem Cells, School of Medicine, University of Nottingham and Nottingham University Hospitals NHS Trust, City Hospital, Nottingham, UK; 6 Department of Clinical Pathology, University of Melbourne, Parkville, VIC Australia

**Keywords:** Breast cancer, Cancer genetics

## Abstract

Intraductal papillomas (IDP) are challenging breast findings because of their variable risk of progression to malignancy. The molecular events driving IDP development and genomic features of malignant progression are poorly understood. In this study, genome-wide CNA and/or targeted mutation analysis was performed on 44 cases of IDP, of which 20 cases had coexisting ductal carcinoma in situ (DCIS), papillary DCIS or invasive ductal carcinoma (IDC). CNA were rare in pure IDP, but 69% carried an activating *PIK3CA* mutation. Among the synchronous IDP cases, 55% (11/20) were clonally related to the synchronous DCIS and/or IDC, only one of which had papillary histology. In contrast to pure IDP, *PIK3CA* mutations were absent from clonal cases. CNAs in any of chromosomes 1, 16 or 11 were significantly enriched in clonal IDP lesions compared to pure and non-clonal IDP. The observation that 55% of IDP are clonal to DCIS/IDC indicates that IDP can be a direct precursor for breast carcinoma, not limited to the papillary type. The absence of *PIK3CA* mutations and presence of CNAs in IDP could be used clinically to identify patients at high risk of progression to carcinoma.

## Introduction

**Fig. 1:**
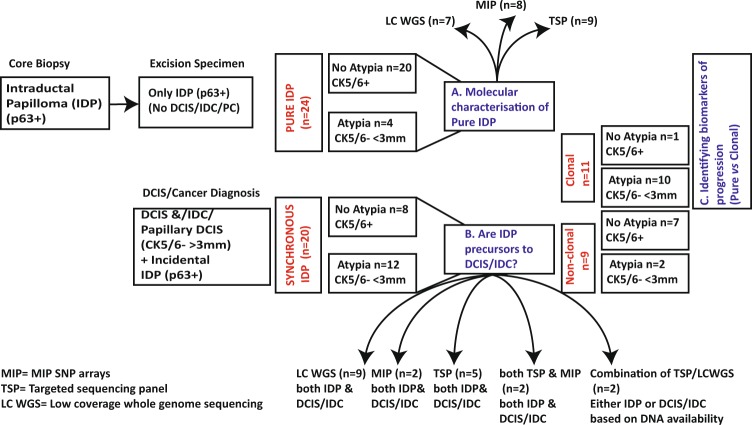
Description and definition of the intraductal papilloma (IDP) cohort. IDP were detected either in core biopsy samples or as incidental findings in excision specimens of breast carcinomas. p63 was used to distinguish between an IDP (p63+) and encapsulated/solid papillary carcinoma (PC: p63−). CK5/6 staining was used to evaluate the extent of atypical cells within IDP. If <3 mm CK5/6-, the IDP was classified as having atypia (i.e. papilloma with ADH, according to WHO guidelines). If the atypical populations were >3 mm CK5/6-, then this was referred to as papilloma with DCIS (i.e. papillary DCIS). We firstly undertook molecular characterisation of pure IDPs (Aim A), which were ascertained based on only a diagnosis of IDP without any association of DCIS/IDC/PC. To investigate the precursor relationship of IDPs with ductal or papillary in situ/cancer, the “synchronous” IDP cohort was investigated (Aim B). These cases were ascertained based on a carcinoma diagnosis in which IDPs were incidental findings. In (Aim C), we compared pure IDP with those synchronous IDP that we found to be clonally related to the coexisting carcinoma, in order to identify biomarkers of progression. Example of the cases were shown in the Supplementary Figs. (1: pure, 3: synchronous). The analytic platforms used are indicated (for details: See Method).

Intraductal breast papilloma (IDP) is characterised by a continuous myoepithelial cell layer with the presence of fibro-vascular papillary stalks and the ductal epithelium^1^. IDPs are a common finding on percutaneous biopsy of screen-detected abnormalities^2,3^ and can occur either without atypical ductal hyperplasia (ADH) (i.e. benign) or with ADH^4^. In this study, IDP with ADH is referred to as “atypical IDP” when there is ≤3 mm of atypical cell populations^5,6^ (Fig. 1). IDP has never been characterised at the molecular level in detail. There are only a few low resolution cytogenetics studies evaluating copy number alterations (CNA) in benign IDP (not associated with carcinoma)^7–11^ but none included atypical IDP. Focused somatic mutation studies have showed a high prevalence of *PIK3CA* mutations and *AKT1* pathway activation in both benign and atypical IDP^12,13^, but interestingly not in papillary carcinoma (PC). These findings raise a question about where IDP fits in the breast cancer progression pathway. Although a diagnosis of IDP carries an increased risk of developing breast cancer, it has long been suggested that IDP could only directly progress to PC. However, it is still inexplicable why IDP has been observed to co-exist with the more common non-papillary forms of ductal carcinoma in situ (DCIS)/ invasive ductal carcinoma (IDC)^14–18^.

In addition to their unknown biology, recent studies suggested that the rate of developing subsequent ipsilateral breast cancer following diagnosis of atypical IDP is higher (13.2% in 8 years) than after benign IDP (5.8%)^4,5,19^. In addition to this significant subsequent risk of future breast cancer associated with IDP, many diagnoses of IDP in a core biopsy are subsequently found to include DCIS or IDC upon surgical excision. These “upgrades” can be as high as 37% for a core biopsy-based diagnosis of atypical IDP and 7–19% for benign IDP^14,19–24^. Although treatment options may vary among clinicians, omission of surgery for asymptomatic IDPs is desirable. However, this goal would require a reliable biomarker for predicting progression.

A detailed understanding of the genetic drivers of IDP might provide an insight into molecular features of progression to ultimately aid in clinical management. Therefore, in this study we performed genetic analysis of IDP cases without carcinoma (“pure” IDP: both benign and atypical IDP) and IDP with coexisting carcinoma of different grades (“synchronous” IDP; ascertained based on cancer diagnosis with IDP being an incidental finding) (Fig. 1).

## Results

### Sample characteristics and architectural features of pure papilloma cases

All pure IDP cases (IDP without coexisting carcinoma) (*n* = 24, Supplementary File 1) were ER+ with a mosaic staining in benign cases but diffuse strong nuclear staining seen in areas with atypia. p63 staining to highlight the myoepithelial cells at the epithelial-stroma interface, which is commonly used to confirm a diagnosis of IDP, was present in all cases. The pure IDP cohort was enriched with benign IDP (*n* = 20) compared to atypical IDP lesions (*n* = 4). Atypical IDP lesions were confirmed as CK5/6 negative in the atypical proliferative areas, which were reconfirmed to be <3 mm of atypia (e.g. Supplementary Fig. 1). Detailed features of benign and atypical IDP lesions are shown in Supplementary File 1. The median follow-up time was 10 years (5–21 years) with no patients recording a subsequent carcinoma diagnosis in that period. The median age of the patient cohort was 60 (range 20–76). Based on the DNA availability, different platforms, either low-coverage whole-genome sequencing (LC WGS), Affymetrix Molecular Inversion Probe (MIP) SNP arrays or a targeted sequencing panel was chosen to identify CNA (Fig. 1). All of these platforms have been previously validated to generate concordant CNA profiles^25^.

### Genetic features of pure papilloma are distinct from ductal or papillary carcinomas

**Fig. 2:**
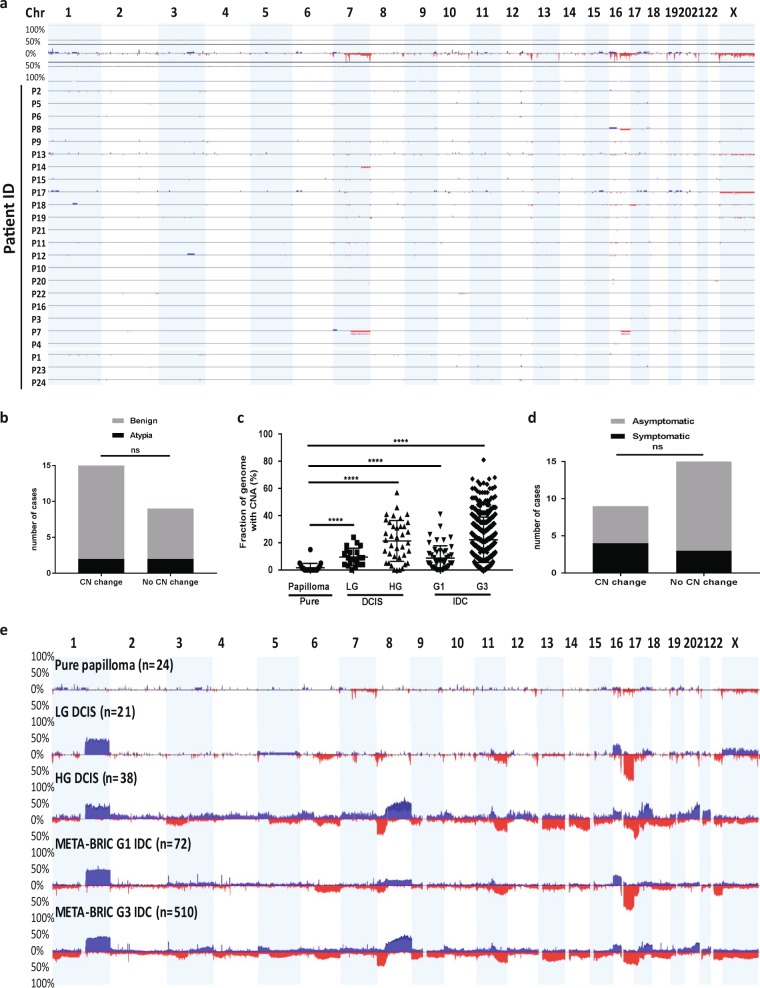
CN analysis of pure papilloma and comparison to carcinoma. **a** Overall CNA profile of 24 pure papilloma cases. Top: Chromosome number from 1–22 and X. Y Axis: frequency of CN changes of all 24 cases. Bottom: Individual sample profiles. Blue = Gain; Red = loss. Purple = allelic imbalance (MIP array samples only). **b** Number of cases of pure papilloma that showed CN change or no CN change based on the histopathological subtype of papillary lesions, benign or atypical. Fisher Exact test was performed, ns not significant. **c** Fraction of the genome altered by CN (%). Unpaired *t*-test with Welch’s correction was used for statistical analysis, ****p < 0.0001. Error bars indicate mean and standard deviation. **d** Number of cases of pure papilloma that showed CN change or no CN change based on whether their diagnosis was symptomatic or asymptomatic. Fisher Exact test was performed, ns not significant. **e** Frequency plots of CN gain (blue) and loss (red) of all cases of pure papilloma, LG DCIS, HG DCIS, IDC (META-BRIC) G1 and G3. **a**, **e** CNA <1 Mb have been filtered out to reduce background noise and sequence artefacts for both IDP and LG DCIS cases.

The CNA observed in the pure IDP cohort (*n* = 24) are summarised in Table 1 and in Fig. 2a. A minority of IDP (37%, 9/24 cases) had CNA and when observed, these predominantly involved a single CN event (6/9 cases, 67%). There was no difference in the presence of any CNA between benign (7/20 cases) and atypical IDP (2/4 cases, *p* = 0.99, Fisher Exact test, two-sided, OR 1.86, 95% CI 0.21–16.18; Fig. 2b) and therefore the data for these were combined for subsequent analyses.

**Table 1:** Summary of genetic features of pure papilloma, clonal papilloma, LG DCIS, HG DCIS and papillary carcinoma.

	Number of cases (%)				Number of cases (%)			*P*-value
Overall CNA (>5 Mb) of pure papilloma	Pure papilloma (*n* = 24)	LG DCIS (*n* = 21)^a^	HG DCIS (*n* = 38)^a^	G1 PC (*n* = 13)^b^	Clonal papilloma with LG carcinoma (*n* = 6)	Clonal papilloma with IG carcinoma (*n* = 3)	Clonal papilloma with HG carcinoma (*n* = 2)	Pure papilloma vs Clonal papilloma
Number of CN changes								
0	15/24 (63%)	1/21 (4.8%)	3/38(8%)	0	0	0	0	**0.0005** Chi-square test
1	6/24 (25%)	1/21 (4.8%)	1/38 (2.6%)	2/13 (15%)	3/11 (27%)	1/11 (9%)	0	
2	2/24 (8%)	8/21 (38%)	1/38 (2.6%)	9/13 (69%)	0	0	0	
3–4	0	4/21 (19.1%)	4/38 (10.5%)	0	2/11 (18%)	1/11 (9%)	0	
>4	1/24 (4%)	7/21 (33%)	29/38 (76.3%)	2/13 (15%)	1/11 (9%)	1/11 (9%)	2/11 (18%)	
CNA (>5 Mb) of pure Papilloma								
16q loss	2/24 (8%)	17/21 (80.9%)	12/38 (31.6%)	21/26 (81%)^b^	7/11 (64%) (4 LG, 2 IG, 1 HG)			**0.001**
16p gain	1/24 (4%)	7/21 (33.3%)	9/38 (23.7%)	N/A	2/11 (18%) (1LG, 1HG)			0.2
Both 16p gain and 16q loss	0	7/21 (33.3%)	6/38 (15.8%)	N/A	2/11 (18%) (1LG, 1 HG)			0.09
X loss^c^	3/24 (13%) (mostly WC)	4/21 (19%) (all partial)	8/38 (21.1%) (mostly WC X)	0	1/11 (9%) (HG)			1
7q loss (partial/full arm)	2/24 (8%)	0	2/38 (5.3%)	N/A	1/11 (9%) (LG)			1
17p loss	1/24 (4%)	3/21 (14.3%)	23/38 (60.5%)	2/13 (15%)^b^	0%			1
10q loss (partial)	1/24 (4%)	0	5/38 (13.2%)	N/A	1/11 (9%) (IG)			1
12q gain (partial) (21.2–21.32) (6 Mb)	1/24 (4%)	0	7/38 (18.4%)	N/A	2/11 (both HG)			0.2
1p gain^c^	1/24 (4%)	0	3/38 (8%)	N/A	0%			1
6p gain (partial) (6p23–21.1)^c^	1/24 (4%)	1/21 (4.8%)	8/38 (21%)	N/A	0%			1
15q gain (partial) (22.2–24.3)^c^	1/24 (4%)	0	4/38 (10.5%)	N/A	0%			1
17q gain (partial)^c^	1/24 (4%)	4/21 (19%)	18/38 (47.4%)	2/13 (15%)^b^	2/11 (18%) (1IG, 1HG)			0.2
19p gain^c^	1/24 (4%)	2/21 (9.5%)	3/38 (8%)	N/A	0%			1
19q gain^c^	1/24 (4%)	1/21 (4.8%)	9/38 (23.6%)	N/A	0%			1

*P*-value from Fisher Exact test (significant P values are in bold) unless specified.

*CNA* copy number alterations, *WC* whole chromosome. *N/A* data not available.

^a^Previously published studies^26,27,39^.

^b^Studies are done by FISH from previous studies^7–9^.

^c^CNA found in exceptional pure papilloma case (P17) with 7 CN events.

The mean fraction of the genome altered (FGA) was 1.6% (range 0–15%; Fig. 2c). The most prevalent CN changes were chromosome X loss (partial or full; 3/24 cases, 13%), followed by 16q loss (2/24 cases, 8%), and 7q loss (partial/full arm; 2/24 cases, 8%) (Table 1). CN change was observed in both symptomatic and asymptomatic IDP (*p* = 0.36, Fisher Exact test; two-sided, OR 0.31, 95% CI 0.05–1.94, Fig. 2d). CN change was not associated with patient age (*p* = 0.11, Mann-Whitney test).

As it has long been thought that benign IDP could progress to only PC due to the similar “papillary” morphology, the CNA data of pure IDP was compared with existing Fluorescence in situ hybridisation (FISH) data for PC^7–9^. The spectrum of CNA of G1 PC reported in these studies is summarised in Table 1. Concomitant 16q loss and 1q gain were the most common events in PC (9/13 cases, 69%), which was very different to the IDPs (8% 16q loss, 0% 1q gain).

The spectrum and frequency of CNA in the pure IDP cohort were also compared with previously published data from LG DCIS, HG DCIS^26,27^, grade 1 (G1) and grade 3 (G3) IDC (METABRIC)^28^ (Fig. 2e, Table 1). Lower CNA frequencies were observed in pure IDP compared to DCIS and IDC of both low and high grades (Fig. 2c, *p* < 0.0001). CNA common to either LG or HG carcinoma were only infrequently observed (e.g. 16q loss, 17p loss). Other CNA recurrently detected in IDP were relatively rare in DCIS/IDC (e.g. 7q loss, X loss) (Table 1).

While pure IDP, unlike breast carcinoma, does not appear to be strongly driven by CN change, one pure benign IDP case was an exception (P17). The CNA observed in this case included gains of 17q, 19q, 19p, 6p, 1p and 15q as well as the loss of chromosome X, most closely resembling a HG carcinoma. This symptomatic case with nipple discharge had no atypia present and had not progressed to carcinoma following excision during 7 years follow-up.

### Somatic mutations in IDP

**Fig. 3:**
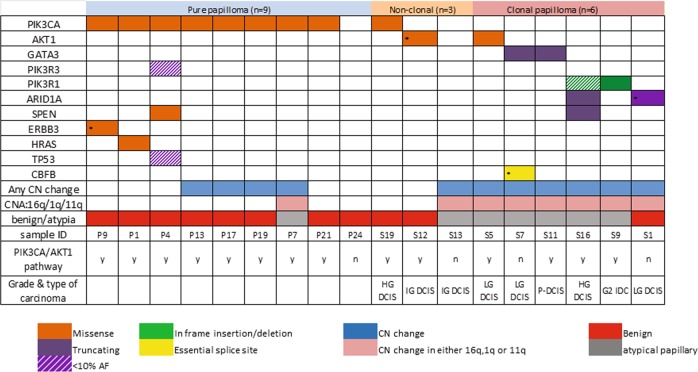
Somatic mutations in papilloma. Mutations identified by targeted sequencing panel in pure papilloma (*n* = 9), non-clonal papilloma (*n* = 3) and clonal papilloma (*n* = 6). Any CN changes as well as change only in either 1q gain, 16q loss or 11q loss along with histopathological subtype of papilloma are shown. *Validated by Sanger sequencing.

Cases of pure IDP with sufficient DNA available (*n* = 9/24) were sequenced using a targeted gene panel (258 genes^27,29^) (Supplementary Table 1) to identify driver somatic mutations (Fig. 3). Pure IDP displayed a low mutation burden with a median of 1 somatic mutation (range 0–4). The most commonly mutated gene was *PIK3CA* with 8 out of 9 (89%) IDP cases harbouring known activating missense mutations. Exons 9 and 20 of *PIK3CA* were sequenced in an additional four cases of pure IDP by Sanger sequencing, which identified one additional mutation making a total of 9 out of 13 pure IDP cases (69%) (8/12 benign and 1/1 atypical; Fig. 3).

No other recurrent somatic mutations were found in the pure IDP cohort; however, single cases had known cancer hotspot mutations in *ERBB3* (Supplementary Fig. 2) and *HRAS*, and a missense mutation in *SPEN* (Fig. 3). One case (P4) carried both *PIK3R3* and *TP53* variants, both with low allelic frequencies (<0.1); however, these could not be validated by Sanger sequencing due to unavailability of DNA. Interestingly, this case also carried *PIK3CA* and *SPEN* variants at higher allele frequencies (0.17, 0.52, respectively, Supplementary Table 2), suggesting that a subclone(s) had arisen carrying the *PIK3R3* and *TP53* mutations.

### Clonality analysis of IDP synchronous with carcinoma

The clonal relationship between 20 IDP cases and synchronous carcinoma (Supplementary File 1, Supplementary Fig. 3) were explored using CNA profiles (*n* = 20) and targeted gene panel sequencing where sufficient DNA was available (*n* = 9, Fig. 3). Details of the cases are summarised in Supplementary File 1. The synchronous carcinomas were divided into LG (LG DCIS), intermediate grade (IG) (IG DCIS/grade 2 (G2) IDC) or HG (HG DCIS/G3 IDC). All synchronous carcinomas were ER+, and three carried an *ERBB2* amplification (one papillary DCIS with mucinous carcinoma and two HG DCIS/IDC). The histopathology of the carcinomas were of no special type, apart from one mucinous invasive carcinoma, and a papillary DCIS (in the same case, S11) (Supplementary File 1).

**Fig. 4:**
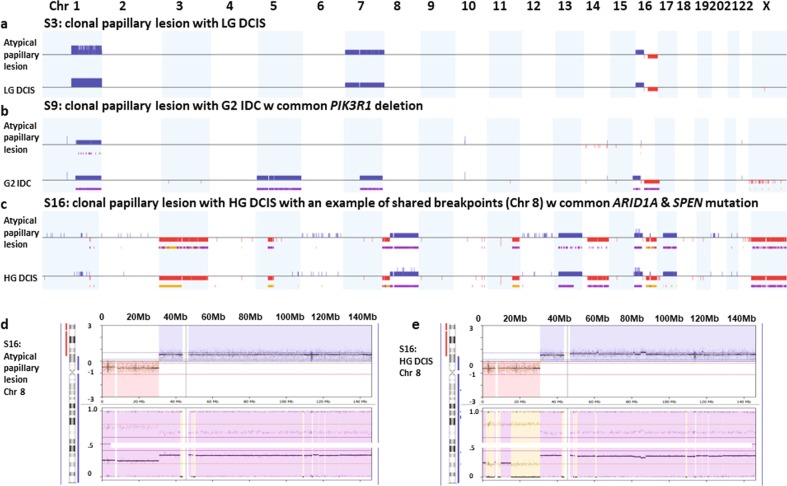
Example of CN changes of clonal papilloma. **a** Clonal papilloma case with LG DCIS (S3). **b** Clonal papilloma case with G2 IDC (S9). **c** Example of a clonal papillary lesion case with HG DCIS (S16) as well as particular locations of shared breakpoint and the same gain of chromosome 8q between the atypical papillary lesion (**d**) and HG DCIS (**e**). CN gain is indicated by blue and loss is indicated by red. Purple is allelic imbalance and yellow is loss of heterozygosity.

Overall, 55% of the IDP (11/20 cases) were clonal with the synchronous cancer. Only 9 out of these 20 synchronous cases have both mutation and CNA data, generated by the targeted sequencing panel (Fig. 1) and the remaining cases have only CNA data derived from either LCWGS or MIP SNP arrays to assess clonality. The majority of cases had at least three shared CNAs, with or without shared point mutations (*n* = 7, e.g. Fig. 4a, Supplementary Fig. 4), or a shared CN breakpoint and shared point mutation(s) (*n* = 2, e.g. Fig. 4b). Two cases where the IDP lesion and LG DCIS shared gain of chromosome 1q with the same breakpoint were also considered clonal (Supplementary Fig. 5). If the breakpoint was not the same we defined this as non-clonal, for example in S13, the papillary component shared 1q gain with the DCIS component, however, without a shared breakpoint (Supplementary Fig. 6). We also considered clonal analysis based on mutations detetcted by the targeted sequencing panel (*n* = 8 pairs) using a previously published method^30^. The clonality index was >0.8 for all cases, reconfirming their clonal relationship (Supplementary Table 3).

**Fig. 5:**
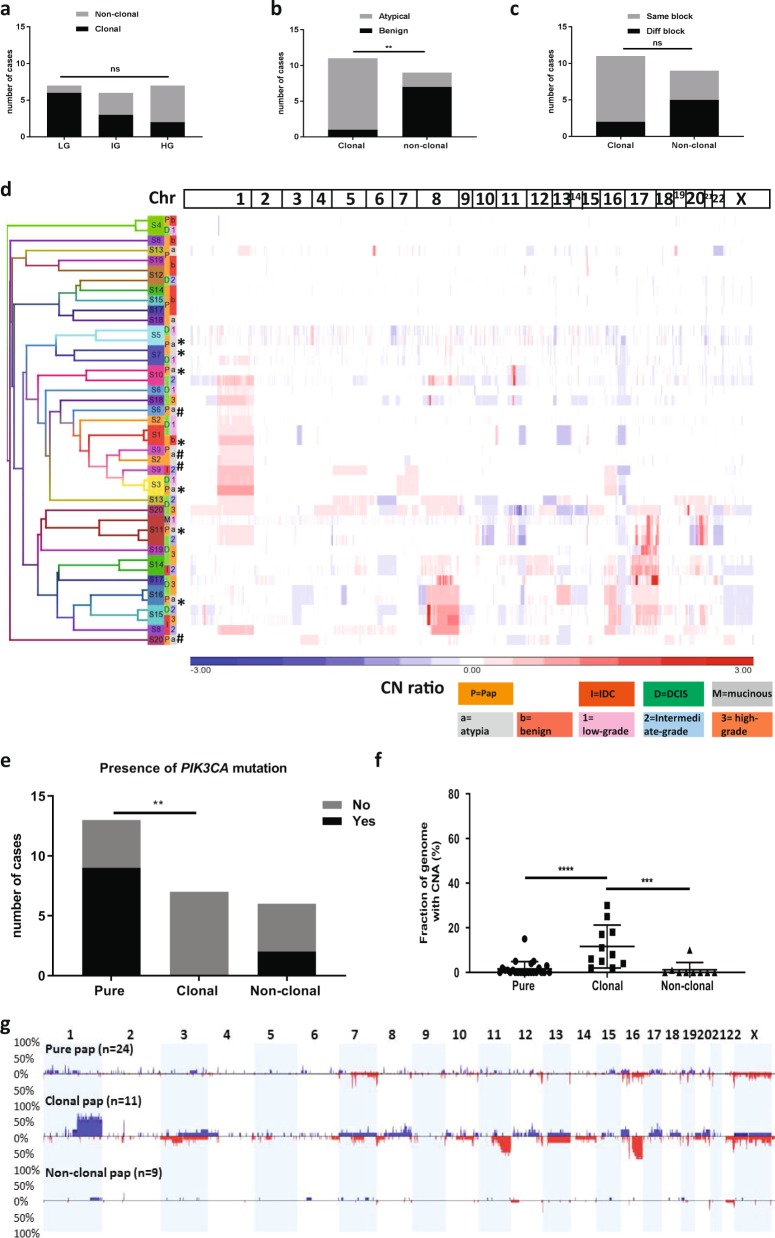
Clonality of papillomas. **a** Number of clonal/non-clonal cases between the three grades of carcinoma. **b** Number of clonal/non-clonal cases between the two histopathological subtypes (benign and atypical papillary lesions). **c** Number of clonal/non-clonal cases based on whether they are in the same/different block. **a**–**c** Fisher Exact test was performed. **p < 0.01, ns not significant. **d** Unsupervised hierarchical clustering of all clonal and non-clonal papilloma cases with carcinoma, based on CNA segments extracted from Nexus. Left: the sample ID and the component of the cases (papilloma=P (orange)/DCIS=D (green)/IDC=I (red)/mucinous carcinoma=M (grey)); the grades of DCIS/IDC: low grade=1, intermediate=2, high grade=3; papilloma: a= atypia/b= benign. Row dendrogram is coloured by individual sample ID regardless of the components (papilloma/DCIS/IDC) (i.e. 1 case=1 colour). *X* axis: Chromosome number on top; blue=loss, red=gain. Clonal papilloma cases that clustered together (*n* = 7) are marked with black asterisks and the remaining 4 clonal papilloma cases are marked with black hashtags. **e** Number of cases with *PIK3CA* mutation in pure, clonal and non-clonal papilloma. Fisher Exact test was performed; **p < 0.01. **f** Comparison of FGA in pure, clonal and non-clonal papilloma cases (Mann-Whitney two-tailed test), Error bars indicate mean and standard deviation; **p < 0.01, ***p < 0.001. **g** Frequency plots of CN gain (blue) and loss (red) in pure, clonal and non-clonal papilloma.

The rate of clonality was similar regardless of the grade of synchronous cancer (LG (6/7 cases), synchronous IG (3/6 cases) and synchronous HG (2/7 cases) carcinoma (*p* = 0.09, Fisher Exact test, Fig. 5a). There was also no difference observed in clonality rate according to tumour type with IDP being clonal and non-clonal with DCIS that was solid, cribriform, comedo or papillary type. However, a clonal relationship was significantly associated with atypical IDP (10/12) compared to benign IDP (1/8) (*p* = 0.0045, Fisher Exact test, two-tailed, OR 35, 95% CI 2.63–465.39, Fig. 5b). This was consistent with the striking difference observed in terms of the type of papillary lesion between the pure and synchronous IDP cohorts. Atypical IDP lesions were significantly more frequent (*n* = 12) than benign (*n* = 8) in synchronous cases (Fisher Exact test, *p* = 0.0045, two-tailed, OR 7.5, 95% CI 1.85–30.34, Supplementary Fig. 7) compared to pure IDP.

For clonal IDP lesions, 2/11 were in a different block, whereas 5/9 non-clonal IDP lesions were in a different block, which was not statistically significant (Fisher Exact test, *p* = 0.16, two-tailed, OR 5.63, 95% CI 0.75–42.36, Fig. 5c). The size of the synchronous cancers was not associated with clonality rate (clonal mean size = 12 mm, non-clonal mean size = 18.75 mm, *p* = 0.61, Mann-Whitney test). There was no significant difference in the percentage of nuclei positive for Ki67 between pure and clonal IDPs (*p* = 0.093, Mann-Whitney test) nor pure and non-clonal IDPs (*p* > 0.99, Mann-Whitney test) (Supplementary Fig. 8).

### Genetic events in clonal and non-clonal papillary lesions

As another means of summarising the clonal relationships, an unsupervised hierarchical cluster analysis was performed, which showed that 7/11 of IDP which were clonal clustered together with their associated carcinoma components (Fig. 5d). For these cases, the CN profiles were the same (3/7 e.g. Fig. 4a, c) or had only a few differences (4/7). The four non-clustering cases carried 2–4 times as many CNA in the carcinoma components compared to the IDP components (e.g. Fig. 4b), which may explain their clustering to different groups. Despite this non-clustering, we contend that these cases are still clonally related due to (1) the precise location of shared breakpoints (cases S2 and S6 shown in Supplementary Fig. 5); (2) shared gain of chromosome 20 and 12, shared loss of chromosome 11 and 13 (case S20, Supplementary Fig. 9); and (3) a shared 1q gain along with a *PIK3R1* deletion (case S9, Supplementary Table 2, Fig. 4b). Overall, 8/11 (73%) clonal cases had at least one additional CNA in the associated carcinoma. In addition, four cases had an additional event in the IDP component, suggesting these IDP lesions kept evolving on their own. For non-clonal cases, the IDP components mostly clustered together as a group due to their collective lack of CNA.

Although most of the synchronous carcinomas were *ERBB2* non-amplified, one *ERBB2* amplified case, S11 (with both IG papillary DCIS and mucinous carcinoma components), was shown by multiple shared CN gains and losses to be clonal with the atypical IDP lesion (Supplementary Fig. 10). While high level amplification of *ERBB2* was evident in the IDP and DCIS components, the mucinous carcinoma component showed only a low level gain of *ERBB2* (validated by SISH), and an additional 13q loss (Supplementary Fig. 10). The CN profiles might suggest that the mucinous component, while sharing the same ancestor, branched out and kept evolving on its own without *ERBB2* amplification.

Eight out of nine non-clonal cases have no CN events. Three out of these eight cases were subjected to targeted gene panel sequencing, and two (S12 and S19) carried readily detectable mutations not shared with the synchronous carcinoma, further validating their non-clonal status (Fig. 3, Supplementary Tables 2 and 3, Supplementary Fig. 2). In contrast, at least one common mutation between IDP and carcinoma components was found in all six clonal cases sequenced on the gene panel, validating their clonality (Fig. 3, Supplementary Table 3, Supplementary Fig. 2). The shared mutations between IDP and carcinoma components are detailed in Supplementary Table 2.

Due to the insufficient DNA for the remaining cases, four cases (three non-clonal and one clonal) were subjected to only *PIK3CA* hotspot mutation assessment by Sanger sequencing of exons 9 and 20. One of the non-clonal cases (S18) had a *PIK3CA* mutation only in the IDP component (p.H1047R). Taken together, *PIK3CA* mutations were found in 2 of 6 non-clonal IDP and 0/7 clonal IDP, compared to 9/13 in pure IDP. There was a significant difference observed in *PIK3CA* mutation status between pure and clonal IDP cases (*p* = 0.006, Fisher Exact test, Fig. 5e).

### Pure IDP lack some CN events frequent in breast carcinoma

The frequency of CN changes of pure, clonal and non-clonal IDP lesions was compared. Non-clonal IDP cases (*n* = 9) showed a significantly lower FGA than pure IDP (Fig. 5f, *p* = 0.04, Mann-Whitney test) with almost no CN change, whereas clonal IDP had a significantly higher FGA than pure IDP (*p* = 0.0002, Mann-Whitney test). The specific CN changes among these three groups were different with a significant enrichment of 1q gain, 16q loss and 11q loss in clonal IDP compared to pure IDP (*p* < 0.001, Fisher Exact test, Fig. 5g, Table 1, Supplementary Table 4).

If the clonal papillomas represent a precursor lesion, we would expect additional genetic events upon progression to carcinoma. Indeed, the frequency of some CNA increased across the spectrum of IDP to LG to HG DCIS/IDC (Table 1, Supplementary Table 4). In particular, 5p gain was observed only in the carcinoma components of three synchronous cases, including whole chromosome 5 gain in two cases. In contrast, gains on 17q, 8q, 1q and loss of 16q, 22q, partial or full arm X, 10q were seen in both clonal IDP and synchronous carcinoma, and are also common alterations in DCIS/IDC.

### Biomarkers for predicting risk of malignant progression of IDP

**Fig. 6:**
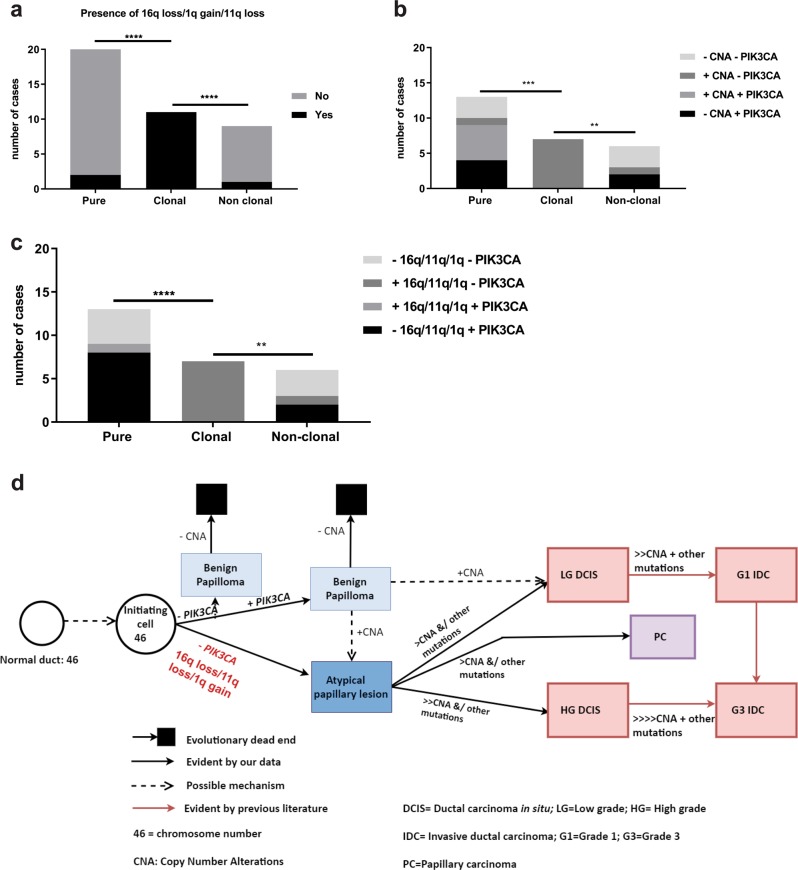
Biomarkers for predicting likelihood of progression to carcinoma and schematic representation of revised breast cancer progression model including intraductal papilloma as a true precursor to both ductal and papillary carcinoma. **a** Number of cases with any of the indicated CN events comparing pure, clonal and non-clonal papilloma. **b** Number of cases showing combinations of any CN change and *PIK3CA* mutation. **c** Number of cases showing combinations of any of 16q loss/11qloss/1q gain and *PIK3CA* mutation. Fisher Exact tests were performed; ****p < 0.0001, ***p < 0.001, **p < 0.01. **d** An unknown event in normal ductal epithelium produces a diploid tumour-initiating cell (46 chromosomes) with a growth advantage that evolves to become benign papilloma. The benign papilloma presumably has a proliferation advantage by often harbouring a *PIK3CA* mutation; however, this is insufficient to continue the clonal expansion for tumorigenesis as shown by “evolutionary dead end” and could stay as benign without further progression. Occasionally benign papilloma could have a selective advantage by gaining specific CNA to progress to atypical IDP lesion/ LG DCIS. Without a *PIK3CA* mutation, the initiating cell could progress towards an atypical papillary lesion directly. Once CN changes in any of 1q gain/16q loss/11q loss occur, these driver alterations provide a selective advantage and progress towards either LG or HG DCIS or PC with subsequent additional CNAs and further mutations. A supportive microenvironment is likely also crucial. Progression towards to HG DCIS from atypical papillary lesions might need a higher level of CNA than progressing towards LG DCIS (shown as the number of “>”).

The existence of higher frequency and type of CNA in clonal IDP than pure IDP cases (Fig. 5f, g) suggest that these are potential genetic events predisposing to progression from IDP to carcinoma. All 11 clonal IDP cases carried either 1q gain, 11q loss or 16q loss compared with only 2/24 of the pure IDP cases (*p* < 0.0001, Fisher Exact test, Fig. 6a) and 1/9 non-clonal IDP cases. Moreover, for the cases with available mutation and CN data, a significant difference was observed between pure and clonal cases with the absence of *PIK3CA* mutation together with the presence of any CN event (Fig. 6b, *p* = 0.0002, Fisher Exact test) or with any of 1q gain, 11q loss or 16q loss (*p* < 0.0001, Fig. 6c). Interestingly, while the clonal IDP components lacked *PIK3CA* activating mutations, 6/11 cases did harbour at least one change that could affect the *PIK3CA/AKT1* pathway including deletion in *PIK3R1* (*n* = 2), *AKT1* mutation (n = 1), CN gain of *PIK3CA* (*n* = 2), loss of *PTEN* (*n* = 1) or even gain of *EGFR* (*n* = 1).

## Discussion

While many clinicians would agree not to treat asymptomatic IDP with surgical excision, the treatment decision still varies. Clinical observational studies showed that IDP may progress to IDC of no special type or cribriform subtypes^4^ or co-exist with DCIS/IDC^14–17^ of various morphologies and grades^14,18^. Here we find that the absence of CN events, regardless of histopathological subtype, symptoms or age of the patient, is strongly associated with lack of progression potential, raising the possibility that IDP showing no CN events could be spared from routine surgical excision.

The finding that most pure IDPs have no CN events is supported by previous locus-specific cytogenetics studies performed by FISH (*n* = 7–12)^7–10^, as well as CGH studies (*n* = 22^11^, and *n* = 5^31^). The key limitations of previous IDP studies are the poor resolution of the methods used, that have been performed only on benign IDP, the lack of p63 staining to validate the diagnosis, the very small sample sizes and the absence of CNA data for synchronous cases. In addition, studies concentrating on somatic mutations in *PIK3CA* and *AKT1* in pure IDP and later carcinomas lacked CNA data^12,13^. In the current study, we combine recent improvements in genomic analysis technology with expert pathological review incorporating p63 staining for a cohort consisting of both pure and synchronous cases. Importantly, careful review for atypical IDP lesions was carried out to distinguish them from high risk cribriform, micro-papillary type ADH as suggested by recent WHO guidelines^6^.

Interestingly, IDP has previously been thought to be a precursor only to PC, although evidence for this was circumstantial and related to similar morphological features and the occasional observation that PC could arise within the fibro-vascular core of benign IDP^4,32^. In one LOH study, 4/11 benign IDP synchronous with PC were clonal^33^. The relationship of IDP with other types of breast cancer is unknown with only a single case of benign IDP synchronous with IG DCIS and mucinous carcinoma being reported to date, which showed that it was non-clonal^11^. The present study definitively demonstrates that a subset of IDP can directly progress to DCIS and IDC of any grade, which extends the finding of a previous study that PC and DCIS share molecular genetic features and likely have a common etiology^34^. Interestingly, our finding of one case where IDP was clonal to both a *ERBB2* amplified papillary DCIS and a HER2-ve G1 mucinous breast carcinoma suggests a broader commonality of molecular etiology and is consistent with recent findings of similarities of mucinous breast cancer and IDC^35^.

In our cohort, atypical IDP was more likely to be clonal with synchronous cancer compared to benign IDP, which confirms clinical data suggesting that atypical IDP lesions are more likely to progress to carcinoma than benign IDP^4,5,19^. Clonal IDP cases could be distinguished from non-clonal and pure IDP by the presence of specific CN events, particularly 16q loss, 1q gain and 11q loss, which are alterations commonly identified in PC^33^ and ductal carcinoma^26,28^. The origin of the carcinomas in cases that were not clonal to the IDPs remain unknown although it is possible that the coexisting lesions do have a common etiology, but through genetic or epigenetic processes not detected in our targeted assay. Additionally, although the synchronous IDP lesions morphologically looked like IDP (p63 positive), we also considered whether the dissected IDP lesions clonal with carcinoma were colonized by the cancer components through the ducts. Since the atypical populations of IDP will be at most 30% of the whole IDP (by definition), if detection of genetic events was derived from the carcinoma component, the log_2_ ratios of shared CNA and the allele frequencies of shared variants would be reduced to at least this proportion in the IDP data. However, such a reduction in log_2_ ratio or allele frequency was not seen.

Our study confirms and extends the findings of previous studies showing that while *PIK3CA* mutation is common in both benign and atypical pure IDPs^12,13^, they are rare in PC or IDC that arise in the context of IDP. It is unclear whether the lack of *PIK3CA* mutations in the clonal IDP/carcinomas is by chance due to the limited cases (*n* = 6) or reflects an intrinsic biological difference in carcinoma arising from an IDP. *PIK3CA* mutation is very common in ductal carcinoma^36^, but as an initiating driver of proliferation in IDPs may lead to an evolutionary dead-end, lacking malignant potential. Weng et al.^37^ also reported that *PIK3CA* mutation in various early breast lesions could provide only an advantage for cellular proliferation, instead of driving towards carcinogenesis, due to the lack of clonal *PIK3CA* mutations in carcinoma components compared to synchronous neoplastic lesions. This paradox remains to be explained, but is supported by the identification of *PIK3CA* mutations, particularly in the kinase domain, in overgrowth syndromes that lack any malignant transformation^38^.

A strength of the current study is the high likelihood that the pure IDP were genuinely low malignant potential lesions because of the absence of subsequent carcinoma even after the long follow-up period (median 10 years). Consequently, the absence of CN events (1q gain/16q loss/11q loss) as well as the presence of *PIK3CA* mutation in IDPs diagnosed in core biopsy irrespective of histopathological features (benign/atypia) or symptoms have the potential to be a biomarker of a low risk malignant potential lesion.

In conclusion, our data suggest a revised model of breast cancer progression, in which IDP, especially atypical IDP lesions, can be a precursor lesion of both LG and HG carcinoma of both ductal and papillary morphology without *PIK3CA* mutation (Fig. 6). The CN state of chromosomes 1, 11 or 16 as well as the *PIK3CA* mutation status of IDPs diagnosed in biopsies could be evaluated further as an assay to predict which subset of patients diagnosed with IDP could have the potential to develop carcinoma. However, the limitation of this study is the small sample size, in particular of pure atypical IDP, which needs to be overcome in the future. In addition, we lack data for cases that have not been surgically excised to assess the natural history of IDP with and without certain genetic events. Our findings may also be relevant to identify women at risk of upgrade to carcinoma when a papillary lesion is detected in a biopsy, although this remains to be tested. Such a prognostic tool might spare the majority of women diagnosed with IDP from unnecessary surgery, which will significantly reduce the treatment cost and associated psychological and physical impact.

## Methods

### Tumour samples

All cases of IDP without carcinoma (pure IDP) and IDP coexisting with carcinoma (DCIS/IDC) (synchronous IDP) were identified from the Royal Melbourne Hospital (RMH) breast service records from 1995 to 2015. Pure IDP cases were all surgically excised as the standard therapy and finalised using data from the Victorian Cancer Registry (to 2017, median 10 years of follow-up) to rule out cases with previous cancer, later development of cancer (>1 year after initial IDP) and cancer on subsequent excision (<1 year after initial IDP) (Supplementary File 1). Synchronous cases were ascertained on the incidental presence of IDP in a primary diagnosis of DCIS/ IDC. Symptoms such as nipple discharge or lump were recorded if available. Cases were considered as asymptomatic when no symptoms (lump/ nipple discharge) were recorded. Archival diagnostic FFPE tumour blocks of pure IDP (*n* = 29) and synchronous IDP with DCIS and/or IDC/Papillary DCIS (*n* = 25) were obtained from the RMH Pathology department. Since these cases were identified by multiple pathologists of different levels of expertise, all cases were subsequently reviewed by an experienced breast anatomical pathologist (P. Hill) and 28/29 pure (Supplementary Fig. 1) and 24/25 synchronous cases (Supplementary Fig. 3) were confirmed as IDP. The criteria for benign/atypical IDP lesion were followed according to Page et al. (i.e. ADH within papilloma) combined with the recent recommendation of the World Health Organisation (WHO) (papilloma with <3 mm extent of ADH)^4,6^. Details of patient selection are summarized in Supplementary Fig. 11. Immunohistochemistry (IHC) staining was assessed in 43/50 cases for p63 and CK5/6 to determine the differential diagnosis between benign IDP and PC (p63 + ve = benign IDP, Supplementary Fig. 1, p63-ve = PC) as well as reconfirming the atypical populations (<3 mm CK5/6 –ve = papilloma with ADH or referred to here as “atypical IDP”, Supplementary Fig. 1; >3 mm CK5/6-ve = papilloma with DCIS), respectively (Supplementary File 1). Cases were excluded for poor staining (n = 2/7) or unavailability of sections (5/7).

Genomic data for LG DCIS cases (*n* = 21) and HG DCIS cases (*n* = 38) was derived from our previously published work^26,27,39^ where cases were obtained from the RMH, the Peter MacCallum Cancer Centre and the Nottingham University Hospitals NHS Trust, City Hospital, Nottingham, UK. Histological review of all these cases was performed by the same pathologist (P. Hill).

This study was conducted under ethical approval from the Peter MacCallum Cancer Centre (HREC #12–64), Melbourne Health (HREC# 2012.119) and the North West-Greater Manchester Central Research Ethics Committee 15/NW/0685. This study was performed under a waiver of consent because of difficulty in contacting women whose diagnosis was >10 years prior.

### Tissue micro-dissection and DNA extraction

IDP and associated cancer tissues were micro-dissected from 8–10 micron haematoxylin and eosin (H&E) stained sections (9–20 sections) either by manual micro-dissection or using the Roche Automated Tissue Dissection System (Roche) to achieve >50% tumour tissue purity. For synchronous cases, tissues were dissected from the IDP lesion, DCIS and/or IDC or Papillary DCIS separately. As benign lesions can disappear or seem different in freshly cut sections, one new reference H&E slide was evaluated by a pathologist (JMP) before tissue dissection to compare to the diagnostic H&E from RMH, marked by PH. Micro-dissected tissue from the Roche Automated Tissue Dissection System was placed in 100 µl of buffer ATL (Qiagen, Hilden, Germany) and then sonicated using the Covaris LE220 system (Covaris, Inc, Woburn, MA, USA), followed by the addition of 20 µL of proteinase K and incubation at 56 °C overnight. DNA was extracted using the MagAttract® HMW DNA mini Kit (Qiagen) as described previously^25^. DNA was extracted from manually dissected tissue using the Qiagen DNeasy Kit as described previously^26^ followed by quantification. Quant-iT^TM^ dsDNA High-sensitivity Assay Kit (Invitrogen, Carlsbad, CA, USA) was used to determine the concentration of extracted DNA. The quality of DNA was assessed by a multiplex PCR assay with primer sets that produce 100–700 bp fragments from non-overlapping target sites in the *GAPDH* gene as described previously^25^ and the quality varied among samples, ranging from 100 bp to 700 bp fragments (Supplementary File 2).

### Sample selection for genetic analysis method

Final case selection is summarised in Supplementary Fig. 11 based on diagnosis, dissection, DNA availability and successful library preparation and good quality data. The first 12 cases with at least 40 ng DNA available were processed for the MIP SNP arrays, but later cases were analysed using a targeted sequencing panel. Cases with a low amount DNA available (5–25 ng) were processed for LCWGS. The method utilised for each case is described in Supplementary File 1 and the DNA input for library preparation for LCWGS/targeted sequencing panel is stated in Supplementary File 2. For pure IDP cases, samples were processed for only the IDP component. For synchronous IDP cases, samples were processed from all IDP lesions and their paired DCIS and/or IDC/Papillary DCIS components. The final number of cases was 24 for pure IDP and 20 for synchronous IDP lesions. All cases were subjected to one of the techniques mentioned above depending on how much DNA was available; except two synchronous cases that were subjected to both MIP and the targeted sequencing panel for both IDP and DCIS/IDC components (Case S9, S16) (Supplementary File 1). The majority of synchronous cases had both components analysed with the same technique, except two for which LCWGS was chosen for the component with low-input DNA and targeted sequencing for the other (Case S11, S12). A somatic mutation found by the targeted panel (Case S12: IDP component) was then validated for its presence in the synchronous DCIS by Sanger sequencing.

### NEBNext® Ultra^TM^ II DNA Library Prep and Low-coverage whole-genome sequencing (LCWGS)

A low DNA input library preparation protocol was used for all samples using the NEBNext® Ultra^TM^ II DNA Library Prep Kit (NEB E7645S/L, New England BioLabs® Inc., Ipswich, MA, USA) as described^25^. In brief, fragmented DNA by Covaris S2 in 50 µL was used for NEBNext End Prep, followed by an immediate adaptor ligation step with a 1.5 µM diluted adaptor. After cleaning up of adaptor ligated DNA, PCR amplification was carried out with eight cycles and 10 cycles for 20 ng and 5–10 ng input, respectively. The mixture of AMPure XP beads and the PCR products were incubated at room temperature for at least 20 min. Subsequently, after ethanol washes, 33 µL elution buffer (0.1 X TE) was added and incubated for 10 min. 2 µL of the final 30 µL library was analysed with the TapeStation (Agilent 2200, Santa Clara, CA, USA) for the size distribution. These libraries were used for LCWGS as described^25^. Briefly, an Illumina Nextseq platform (NextSeq 500) (Illumina, San Diego, CA, USA) (paired-end 75 bp) was used to run the pooled, normalized indexed libraries according to the standard Illumina protocol. The sequencing depth achieved in the samples ranged from 0.72–2.1×. (Supplementary File 2).

### Molecular inversion probe (MIP) SNP arrays

The MIP 330 K OncoScan array was used to analyze pure and synchronous papilloma samples with associated carcinoma components and was performed according to the manufacturer’s instructions by the Ramaciotti Centre for Genomics (version 3, NSW, Australia) or Affymetrix Inc (version 2, Santa Clara, CA, USA). DNA input was 40–100 ng for this assay as described previously^26,40^.

### Targeted sequencing library preparation, enrichment and sequencing

Targeted sequencing of tumour DNA was performed using an Agilent SureSelect Custom Panel targeting 258 genes (total targeted region of 1.337 Mb^27,29^) (Supplementary Table 1) including breast cancer driver genes such as *PIK3CA, AKT1, PIK3R1, GATA3, PTEN, TP53, ARID1A*. The panel is designed based on known breast cancer genes from large cohort studies including TCGA and has previously been used by Lee et al.^29^. Library preparation was performed mostly from an input of at least 100 ng of DNA using the KAPA Hyper system (Agilent, Santa Clara, CA, USA) as described previously^29^ except three pure IDP cases where 40–70 ng DNA was used (DNA input: Supplementary File 2). Sequencing of target-enriched DNA libraries were performed using the Illumina Next Seq 500 generating 75 bp paired-end sequence reads.

The variants identified by targeted sequencing panel were validated by Sanger sequencing where DNA was available. Additional samples were screened for *PIK3CA* variants in exons 9 and 20. Primers for Sanger sequencing were designed in Primer 3 as described previously^27^ (Supplementary File 2: Primers used, PCR conditions). PCR products were sequenced using BigDye Terminator v3.1 (Applied Biosystems) and a 3730 DNA Analyzer (Applied Biosystems) as described previously^27^. Chromatograms were visualized in Geneious v8.1.9 (Biomatters, Auckland, New Zealand).

### Data analysis

For LCWGS, reads were aligned with bwa mem (v0.7.12-r1039) to hg19 (GRCh37) after removal of sequencing primers by cutadapt (v1.7.1) as described previously^25^. ControlFREEC (version 6.7)^41^ was used to estimate copy number from the LCWGS data in 50 kb windows, with default parameters, no matched normal sample and baseline ploidy set to 2 as described previously^25^. To reduce spurious calls, blacklisted regions (problematic regions, including highly repetitive centromeric regions, where DNA copy number cannot be accurately measured) as identified from Scheinin et al.^42^ were filtered out as described^25^.

MIP data were pre-processed by the Ramaciotti Centre for Genomics or Affymetrix Inc., with tumor samples batch normalized against Affymetrix controls as described previously^26^.

For Targeted Sequencing, paired-end sequence reads were aligned to the g1k v37 hg19 reference genome using BWA^43^. Optical duplicate reads were removed using Picard (http://broadinstitute.github.io/picard/) (v1.119), then local realignment around indels and base quality score recalibration were performed using the Genome Analysis Tool Kit (GATK v3.2) in accordance with their recommended best practice workflow^44^. SNP and indel variants were called using GATK Unified Genotyper, Platypus^45^ and Varscan 2^46^. Called variants were additionally annotated using the Ensembl Variant Effect Predictor^47^. Somatic mutations in the tumour sequencing data were identified by applying the following filters: canonical transcript; variants identified by at least two variant callers; minor allele frequency (MAF) present at ≤0.001 in ExAc (Version 0.3.1, excluding TCGA data, released March 14 2016)^48^, GnomAD (Version 2.0, released 27 February 2017), EVS (Version ESP6500SI-V2-SSA137)^49^. Manual inspection of the sequence reads using the Integrative Genomics Viewer (IGV)^50^ were performed before finalizing the somatic mutations. Any false positive variants due to sequencing artefacts were excluded from final analysis.

Off-target sequencing reads were used to generate genome-wide copy number data using CopywriteR^51^ using a 50 kb window and utilising a normal lymphocyte DNA control (NA12878, Coriell Institute) run in the same sequencing batch for the normalisation baseline. All samples passed Quality Score in Nexus. However, 7 pure IDPs had extra noise in the CN profile. The background noise or sequence artefacts or the “waviness” of the CN profile was likely due to the poor quality of FFPE samples^40^. In order to reduce the noise/ artefacts of these samples, they were normalised against a normal DNA from FFPE stroma /matched normal (specified in Supplementary File 1) and CN calls were manually curated. For example, MIP data were manually curated through allelic imbalance information, and spurious calls unsupported by allelic data were removed from downstream analysis.

All sample data were imported into Nexus (v8, BioDiscovery Inc., Hawthorne, CA) and segmented using SNP-FASST. Copy number gains were called if the log_2_ ratio of the segment was >0.15 and losses called if <−0.15.

#### Clonality assessment

Since the overall CNA and mutation levels of pure IDP in our study was substantially lower than DCIS and IDC, lesions were classified as clonally related when sharing at least one breakpoint with the same CNA. The shared breakpoints were observed at 50 kb resolution for sequencing data and at the resolution of the SNP loci for MIP array data (Supplementary Figs. 4, 5 and 10). Visually inspected shared breakpoints were emphasized rather than overall CNAs as suggested by Bollett et al.^52^. If no breakpoint was shared, the case was called non-clonal even if the components had the same CNA (Supplementary Fig. 6). In addition, the clonality indices (CI and CI2) were analysed according to Schultheis et al.^30^ based on mutations from the targeted sequencing panel analyses, for those cases where both IDP and DCIS/IDC components were subjected to targeted sequencing (*n* = 8).

#### Clonality assessment based on mutation profile

The synchronous cases were assessed for their clonality status based on mutations generated by the targeted sequencing panel. The two clonality indices (CI and CI2) were calculated as described in Schultheis et al.^30^. For this analysis, all synonymous and non-synonymous SNVs were included as long as they met the criteria followed by the filtering process mentioned above in data analysis. These indices recognise that shared mutation(s) for a synchronous case may happen by chance based on the frequency of the gene mutation in the TCGA dataset (*n* = 977) (http://gdac.broadinstitute.org/runs/stddata__2016_01_28/data/BRCA/20160128/gdac.broadinstitute.org_BRCA.Mutation_Packager_Oncotated_Calls.Level_3.2016012800.0.0.tar.gz). $${\mathrm{The}}\,{\mathrm{clonality}}\,{\mathrm{index}}\left( {{\mathrm{CI}}} \right){\mathrm{was}}\,{\mathrm{defined}}\,{\mathrm{as}} = \left\{ {\begin{array}{*{20}{l}} {1 - \mathop {\prod }\nolimits_{k = 1}^n f_k,n > 0} \hfill \\ {0,n = 0} \hfill \end{array}} \right..$$

Here, *n* = number of shared mutations in IDP and DCIS/IDC components and *f*_*k*_ = the percentage of breast carcinomas from TCGA dataset harbouring a given mutation. We considered a pair of synchronously diagnosed IDP and DCIS/IDC were clonal if CI > 0.8, as suggested by Schultheis et al.^30^.

Schultheis et al. suggested that CI and CI2 are consistent in their study; CI2 was also calculated in this study as an alternative approach. In this alternative approach, the cutoff was calculated as suggested using the R package ROCR (R v3.6.1). The threshold value was returned as 2.84.

CN segments of all components of synchronous cases derived from Nexus were imported into Partek Genome Suite (Partek Inc., St. Louis, MO, USA) in order to carry out unsupervised hierarchical clustering without any normalisation. Pearson dissimilarity and average linkage was used to generate dendrograms.

CN profiles (pure, clonal and non-clonal IDPs) were compared to cases of DCIS (previously published)^26,27,39^, and IDC cases from METABRIC^28^. CNA segments were exported from Nexus and the percentage of genome altered by copy number in base pairs resolution or the weighted fraction of the genome altered (FGA) was calculated. In brief, FGA was calculated by the summation of the CN change in base pairs for each chromosome and then dividing by the length of that chromosome. The final FGA for a sample was calculated by taking the average of the percentage of CN change across all chromosomes^53^. All data are available as described in the Data Availability statement^54–56^.

### Immunohistochemistry (IHC)

ER, Ki67, CK5/6, p63 immunohistochemistry was performed on all cases by the Peter MacCallum Cancer Centre Anatomical Pathology Department using standard protocols. ER was scored using the Allred system^57^. Ki67 scoring was performed using ImmunoRatio (http://153.1.200.58:8080/immunoratio) (Last accessed on October, 2018)^58^. The basal marker CK5/6 was performed to assess the presence or absence of luminal cell layers of atypical IDP lesion. p63 was performed to assess the presence or absence of the myoepithelial layer of benign IDP^59^. HER2 status was taken from the original pathology report when it was available (IHC/SISH/CISH), or from the CN profile of chromosome 17 based on 17q11.2–17q12 high level of amplification.

### Statistical analysis

Graph Pad Prism v7 (GraphPad, Inc, San Diego, CA,USA) was used to generate graphs and appropriate statistics as indicated in each table and figure. Clonality indices were calculated in R (v3.6.1). A p-value of <0.05 was considered significant.

### Reporting summary

Further information on research design is available in the Nature Research Reporting Summary linked to this article.

## Supplementary information

Supplementary material

Reporting Summary Checklist

## Data Availability

The data generated and analysed during this study are described in the following data record: 10.6084/m9.figshare.11791173. The sequencing datasets generated during this study and supporting the conclusions of this article, are publicly available through NCBI Sequence Read Archive: https://identifiers.org/ncbi/insdc.sra:SRP241968. The molecular inversion probe (MIP) single nucleotide polymorphism (SNP) array datasets generated during this study, are publicly available in Gene Expression Omnibus (GEO): https://identifiers.org/geo:GSE131087. Details of patient samples (de-identified data) are publicly available in Supplementary File 1. Data on DNA input and sequencing performance are publicly available in Supplementary File 2.
