# Supplementary material for: The genetic architecture of breast papillary lesions as a predictor of progression to carcinoma
Source: NPJ Breast Cancer. 2020 Mar 12;6:9. doi: 10.1038/s41523-020-0150-6 (PMC7067788; doi:10.1038/s41523-020-0150-6)
Supplement: Supplementary file 1 — Supplementary material [file 41523_2020_150_MOESM1_ESM.pdf]

Supplementary Information: Kader *et al.* 2020.

Supplementary Figure 1

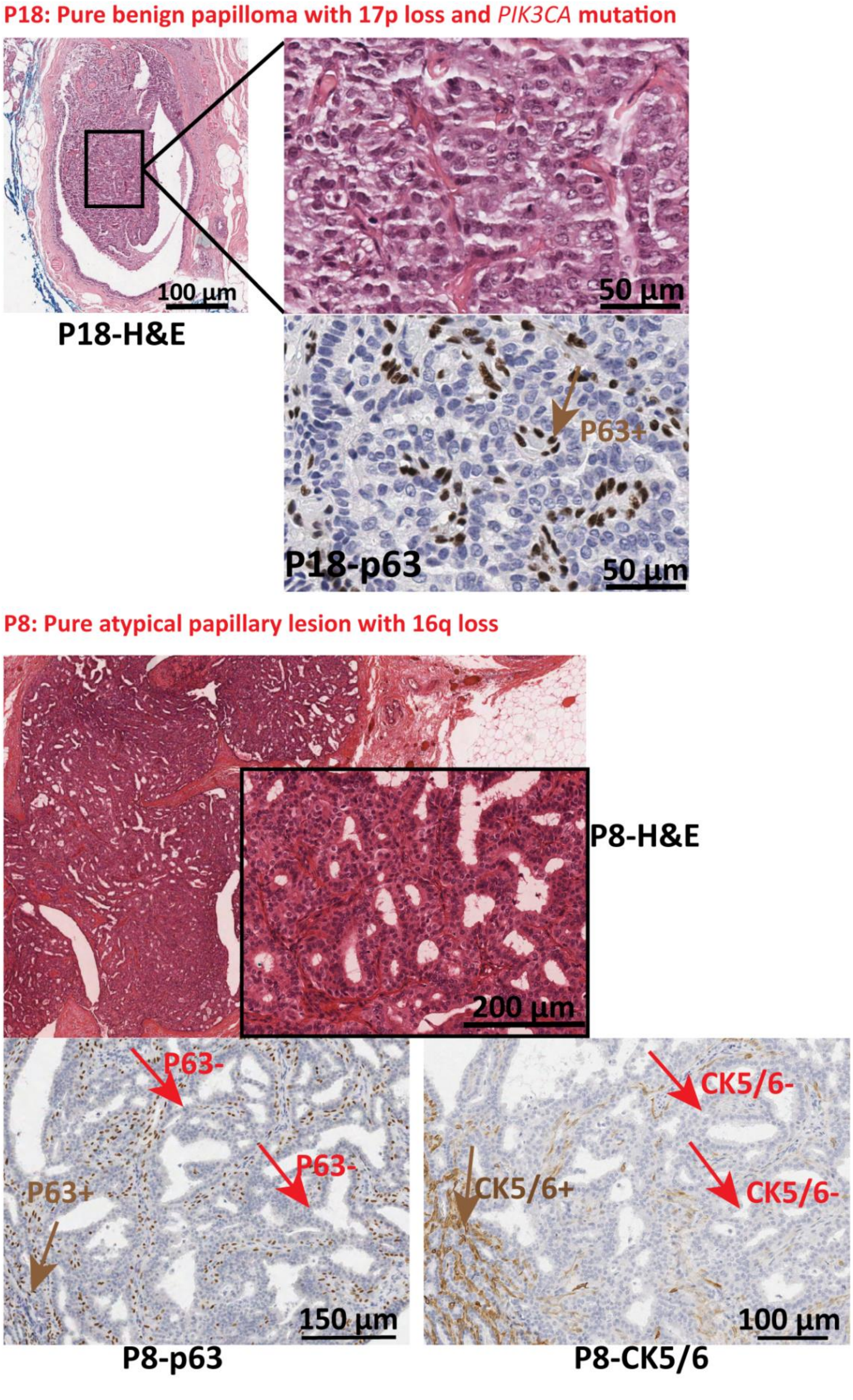

Supplementary Figure 1. Representation of pure papilloma cases. All benign/atypical papillary cases showed p63 positive immunostain (brown staining: brown arrow), suggesting continuous myoepithelial layer and reconfirming them as breast papillary lesions without carcinoma. CK5/6 immunostain was additionally performed only for atypical papillary lesions to reconfirm <3 mm or <30% of populations are atypical/negatively stained (P8: 824.8 µm atypical populations). Atypical populations within this papilloma (Case: P8) are indicated with red arrows (CK5/6-). Areas with CK5/6+ or p63+ are also indicated with brown arrows, suggesting non-atypical areas of this papilloma.

Supplementary Figure 2

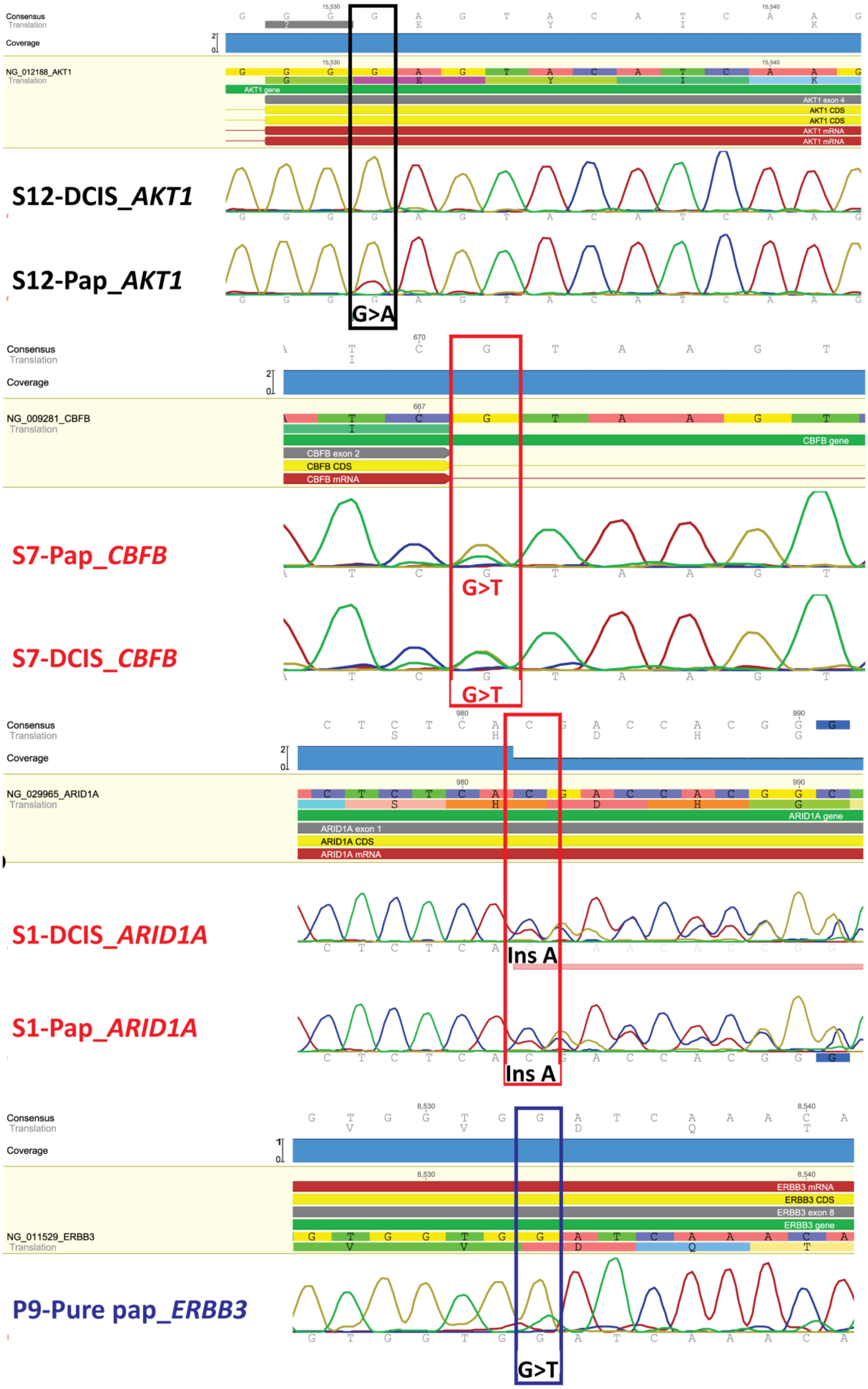

Supplementary Fig 2. Sanger sequencing validation. Validation of mutations identified in *AKT1*, *CBFB*, *ARID1A* and *ERBB3*, by targeted gene panel sequencing.

Supplementary Figure 3

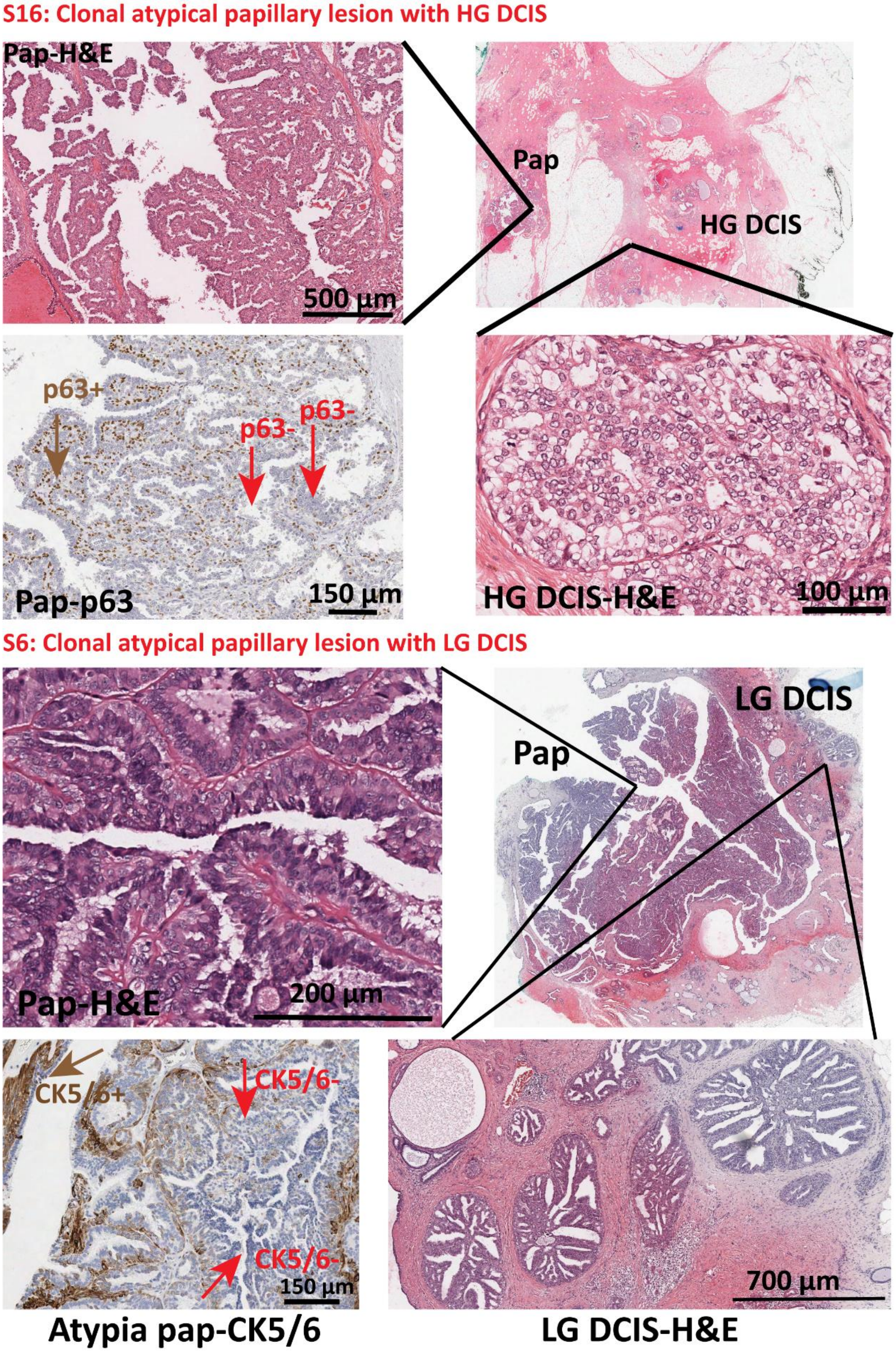

Supplementary Fig 3. Representation of synchronous papilloma with HG and LG DCIS cases. p63 positive immunostain (brown staining) of case S16 suggests a continuous myoepithelial layer and reconfirms this as a breast papillary lesion without carcinoma. Atypical populations within this papilloma are indicated with red arrows. Case S6: Areas with atypical populations are indicated with red arrows (CK5/6-). Non-atypical populations of the same lesion shown as CK5/6+, indicated with brown arrow.

Supplementary Figure 4

Case S16: clonal with HG DCIS: Genome wide CN profile: shared breakpoints

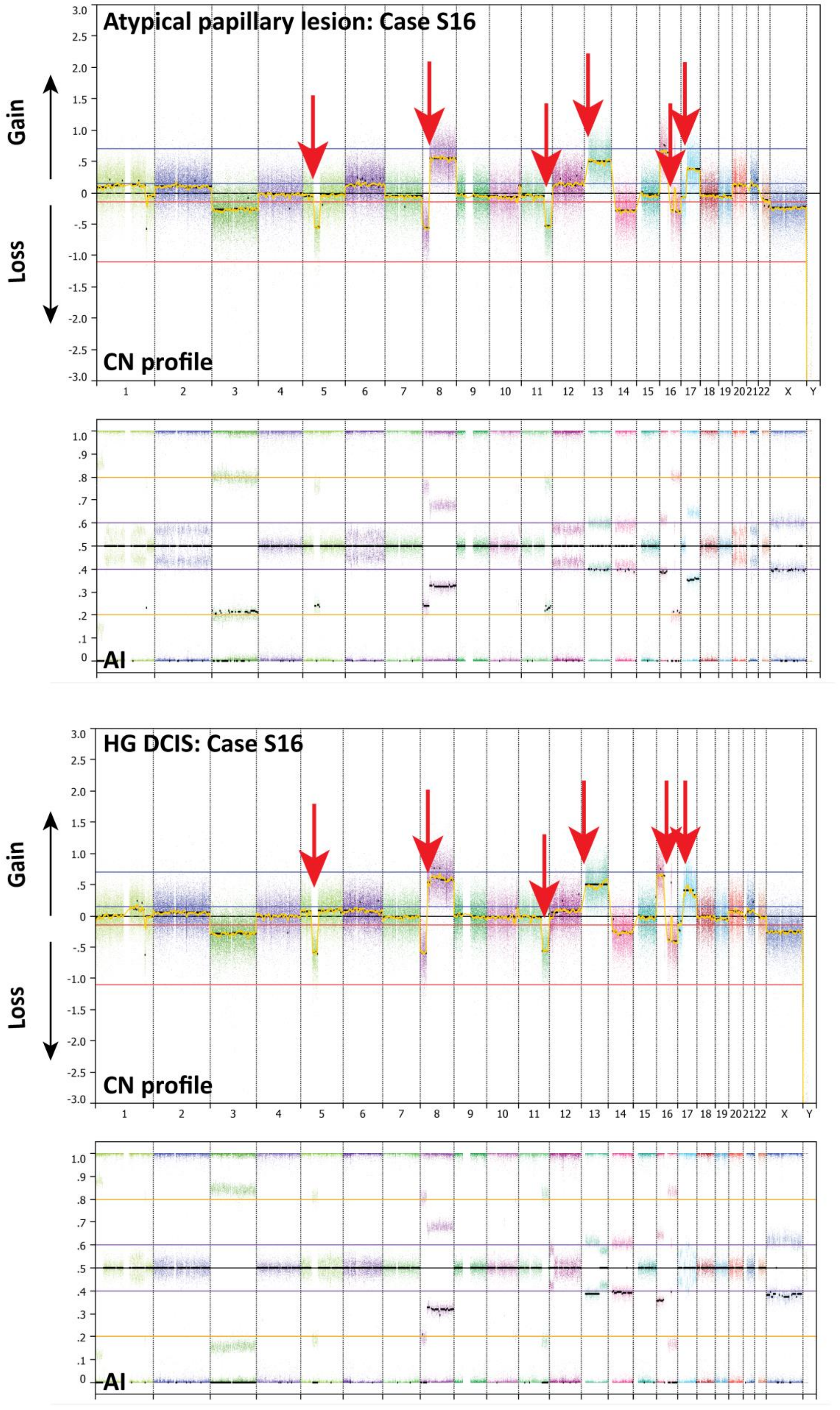

Supplementary Fig 4. Example of shared breakpoints of a clonal atypical papillary lesion with HG DCIS (case S16). Genome wide CN profile of a clonal atypical papillary case (case S16) which showed multiple gains and losses shared with the synchronous HG DCIS. Separate chromosomes from 1 to 22 including X are shown and a $\log_2$ ratio equal to zero corresponds to a copy number of 2. Red arrows indicate the shared break points of these two components. SNP MIP arrays were used for this case. AI= Allelic information.

Supplementary Figure 5

Two clonal cases: non-clustering case in hierarchical cluster analysis

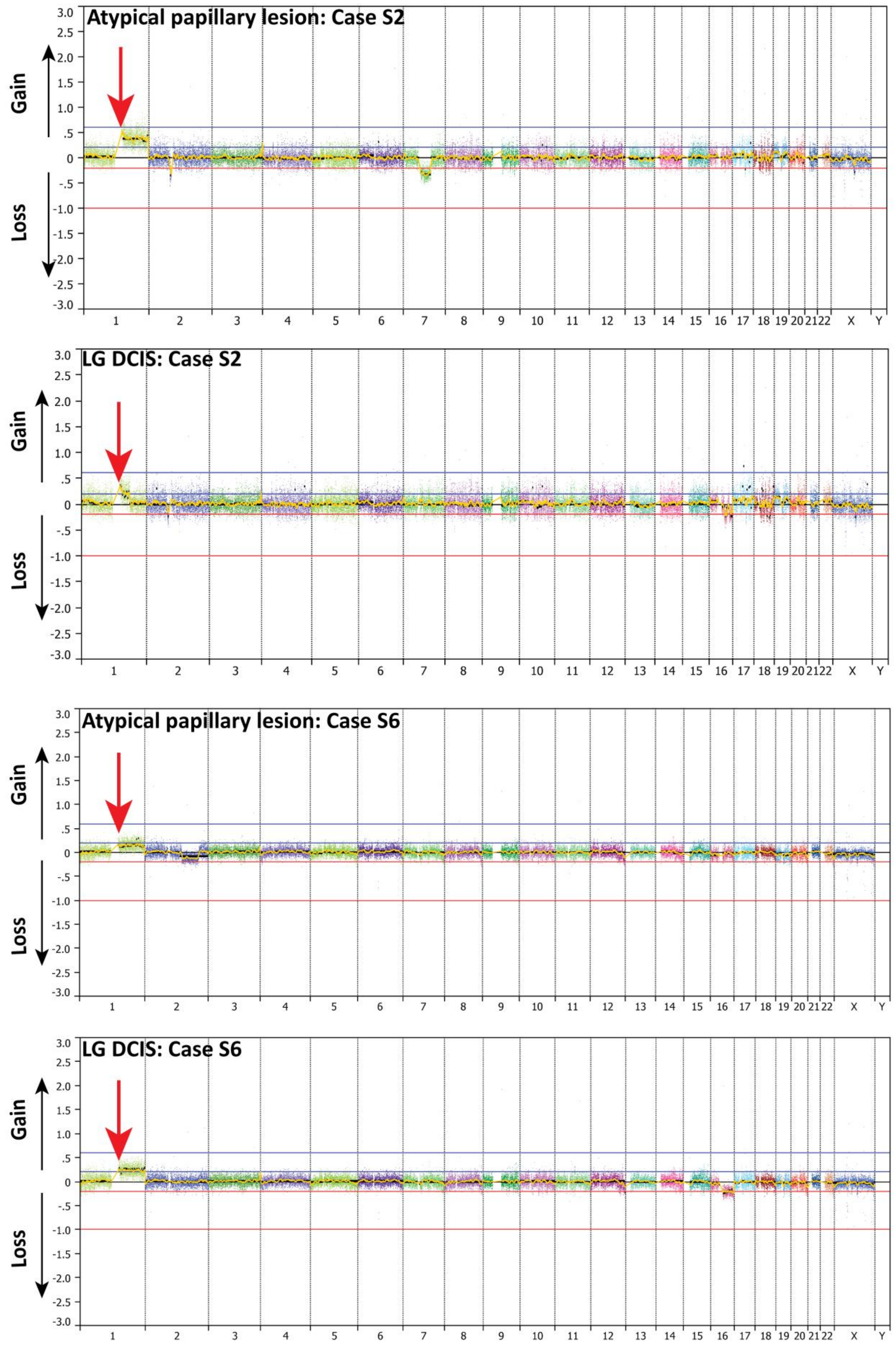

Supplementary Fig 5. Example of shared breakpoints of two clonal atypical papillary lesions with LG DCIS (case S2, case S6). Genome wide CN profile of two clonal atypical papillary cases which did not cluster with their cancer components in unsupervised hierarchical cluster analysis. Separate chromosomes from 1 to 22 including X are shown and a log_2 ratio equal to zero corresponds to a copy number of 2. The profile showed gain of 1q shared with the synchronous LG DCIS for both cases. Red arrows indicate the shared break points of these two components. LC WGS was utilised for these two cases.

Supplementary Figure 6

Case S13: Non-clonal Atypical papillary lesion with IG DCIS:1q gain of papillary component without shared breakpoints with DCIS

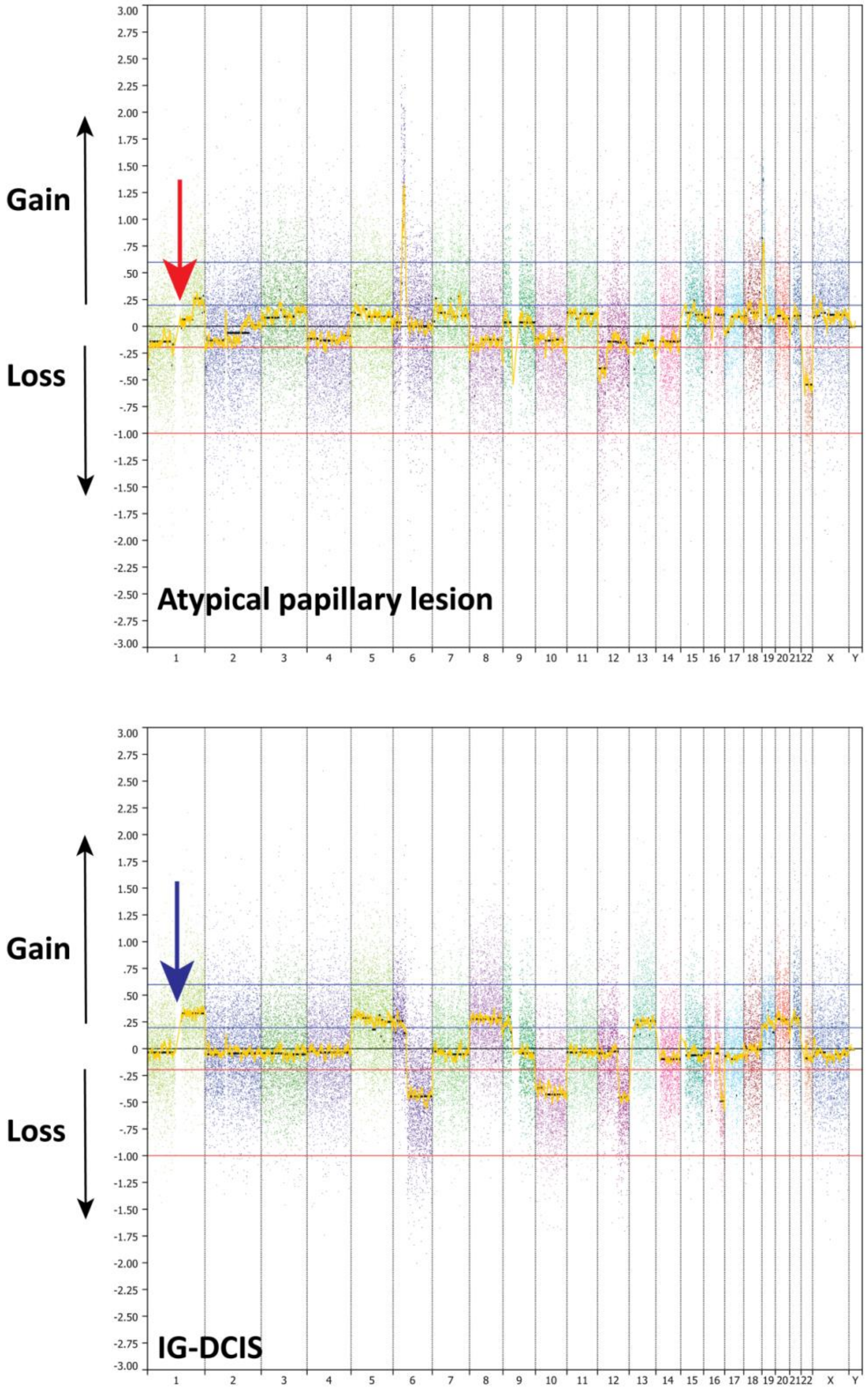

Supplementary Fig 6. Genome wide CN profile of a non-clonal atypical papillary case (S13) which showed gain on 1q without the same shared break points as the synchronous IG DCIS. Separate chromosomes from 1 to 22 including X are shown and a $\log_2$ equal to zero corresponds to a copy number of 2. Arrow indicates break point of 1q gain.

Supplementary Figure 7

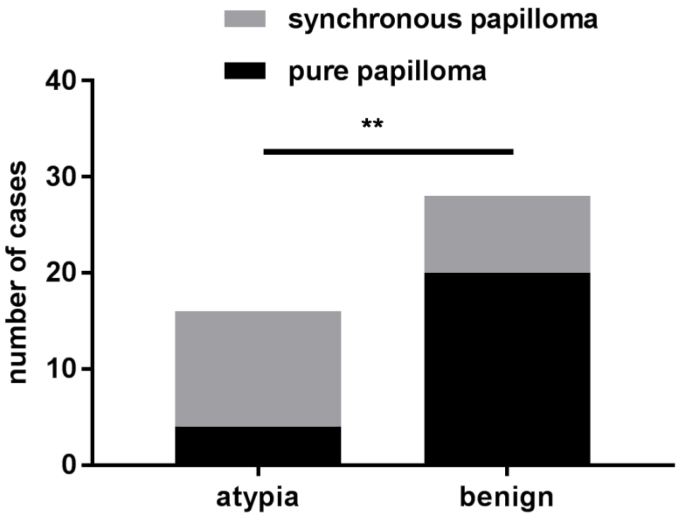

Supplementary Fig 7. Proportion of pure and synchronous papilloma cases with histopathologically classified as benign or atypical. Fisher Exact test was performed, ** p<0.01.

Supplementary Figure 8

A

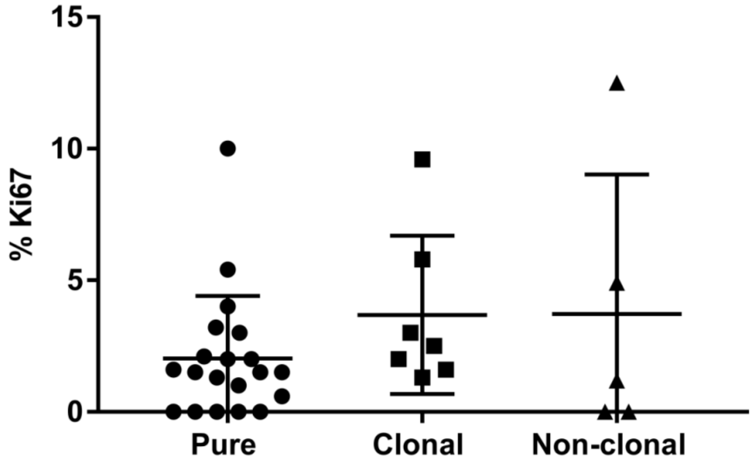

B

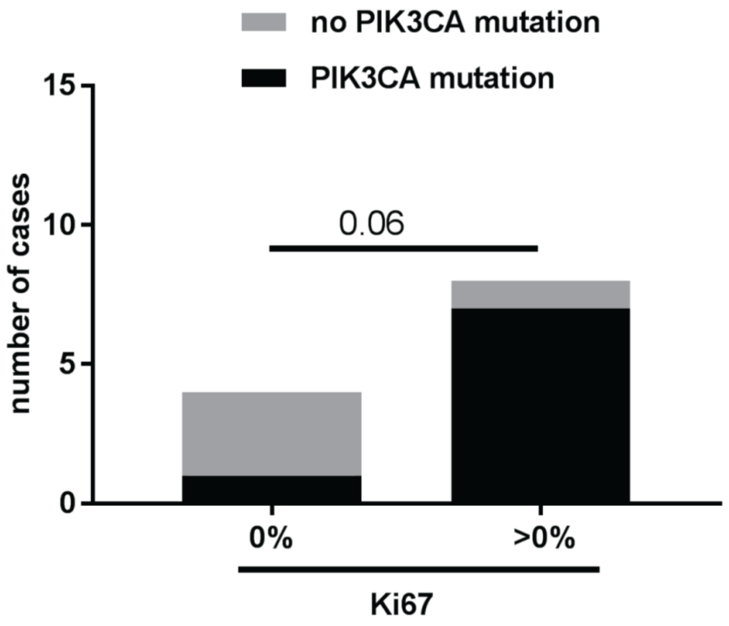

Supplementary Fig 8. Ki67 immunohistochemistry. A. The comparison of percentage of cells showing positive Ki67 immunostaining in pure, clonal and non-clonal papilloma cases. Error bars indicate mean and standard deviation B. Ki67 (% positive cells) and *PIK3CA* mutation status in the pure papilloma cohort.

Supplementary Figure 9

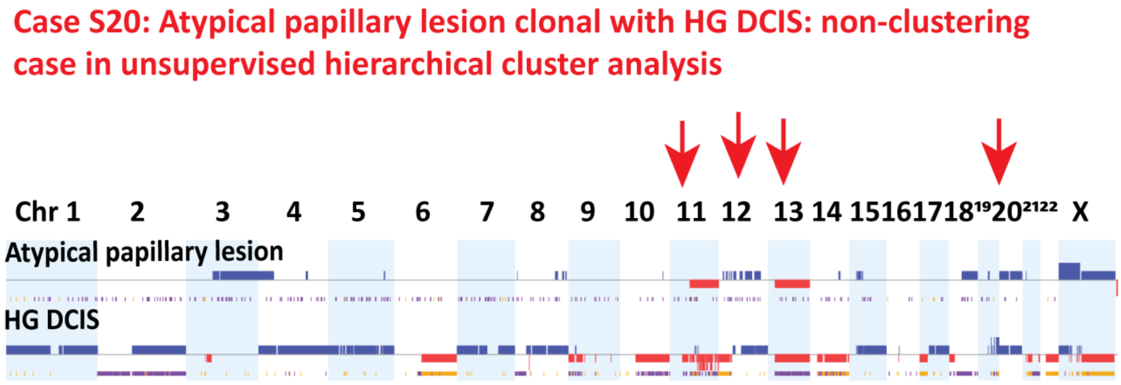

Supplementary Fig 9. Example of a clonal atypical papillary lesion with HG DCIS (case S20). Genome wide CN profile of case S20 which did not cluster with their cancer components in unsupervised hierarchical cluster analysis. Separate chromosomes from 1 to 22 including X are shown. CN gain is indicated by blue and loss is indicated by red. Purple is allelic imbalance and yellow is loss of heterozygosity. Red arrows indicate shared gain of chromosome 20 and 12, shared loss of chromosome 11 and 13 between these two components. SNP MIP arrays were used for this case.

Supplementary Figure 10

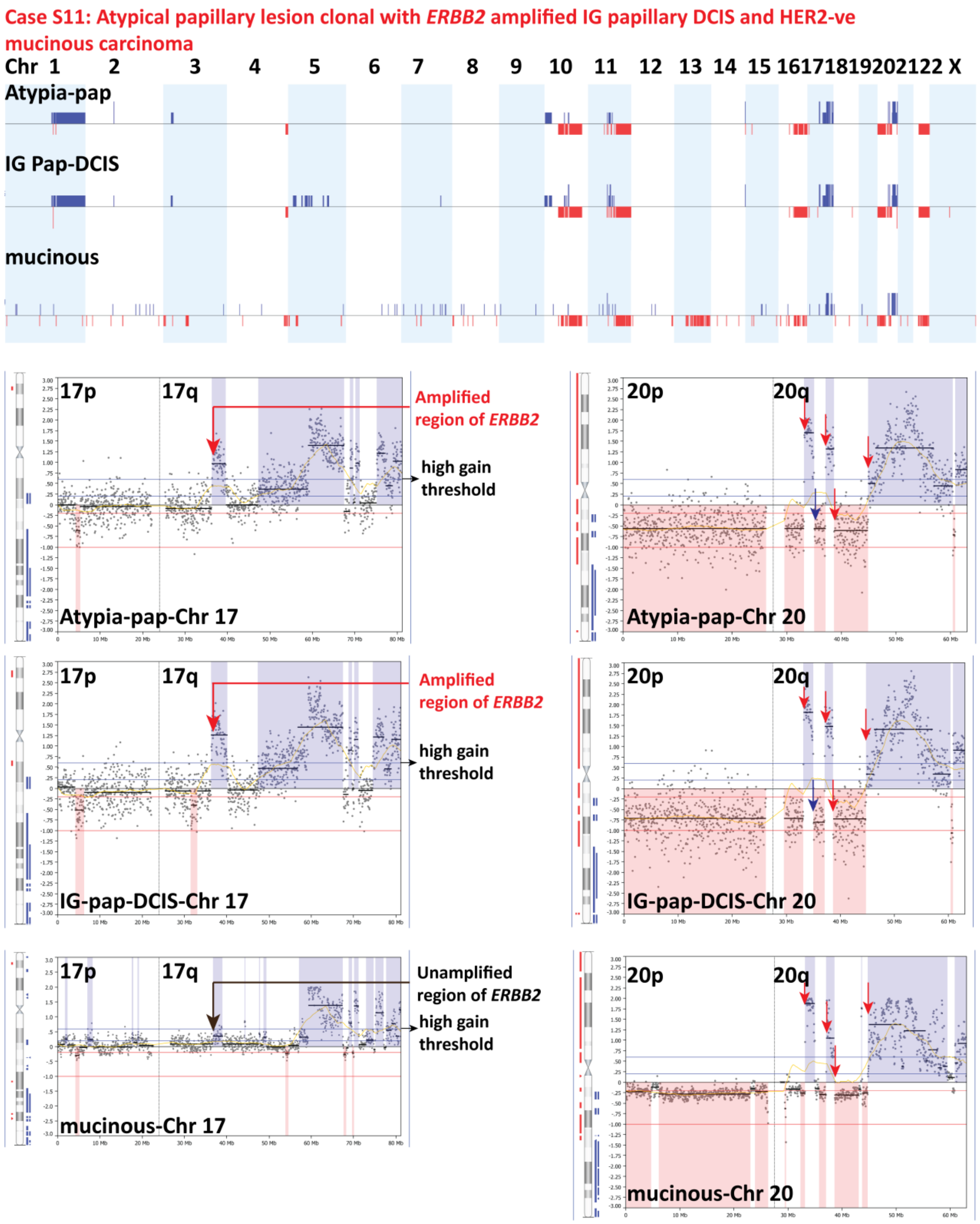

Supplementary Fig 10. Copy number plot of a clonal atypical papillary lesion synchronous with an *ERBB2* amplified IG papillary DCIS lesion and a mucinous carcinoma (HER2-ve). The top panel shows the whole genome wide CN profile of the three components of this case. Blue=gain; Red=loss. The bottom panels show the copy number plot of chromosome 17 and 20 of all components. Amplification of the *ERBB2* region (17q12) (red arrow) was observed in both the papillary lesion and DCIS, however, the mucinous carcinoma showed only low level gain without amplification in that region (black arrow). Chromosome 20 is shown as an example of the shared break points of all components. Red arrow indicates break points are shared in all of the components. Blue arrow indicates shared only between papillary lesion and papillary DCIS components.

Supplementary Figure 11

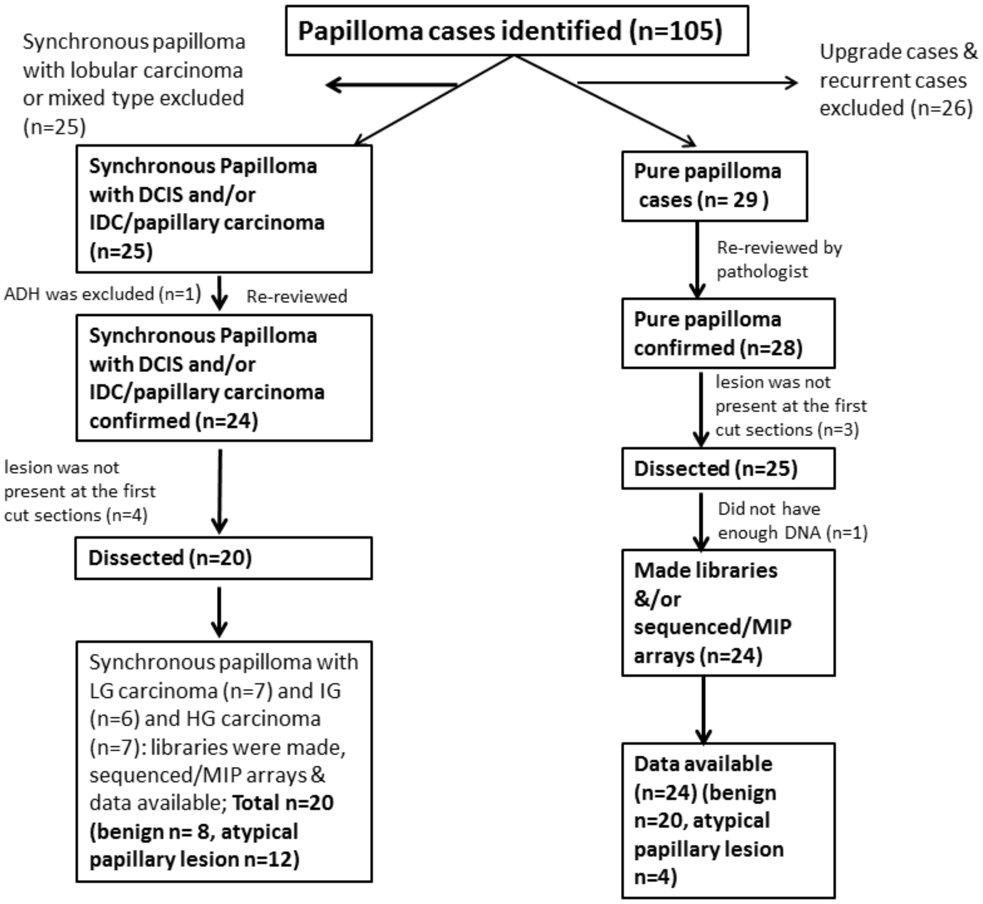

Supplementary Fig 11. Patient selection flow diagram.

Supplementary Tables: Kader *et al.* 2020

Supplementary Table 1. Gene list of Targeted Sequencing panel

ACRBP	*DDB2*	*KCNAB3*	*PARP4*	*THNSL1*
AHNAK2	*DPEP1*	*KCNT1*	*PARPBP*	*TLDC1*
AKT1	*DUSP27*	*KIF27*	***PDGFRA***	*TMCO4*
AKT2	***EGFR***	***KIT***	*PER1*	*TMEM127*
AKT3	*ELL*	***KMT2C***	*PFKM*	*TMTC1*
ALK	***ERBB2***	***KMT2D***	***PIK3CA***	*TNFRSF6B*
ALKBH1	***ERBB3***	***KRAS***	***PIK3R1***	*TNNI3K*
ALKBH2	***ERBB4***	*LAMB1*	***PIK3R3***	*TOX3*
ALKBH3	*ERCC1*	*LIG4*	*PLCD1*	***TP53***
ALMS1	*ERCC2*	*LRWD1*	*PLIN4*	*TPP1*
APC	*ERCC3*	***MAP2K1***	*PLK3*	***TSC1***
APEX1	*ERCC4*	***MAP2K4***	*PLK4*	***TSC2***
APEX2	*ERCC5*	***MAP3K1***	*PMS1*	*UNC45A*
ARID1A	*ERCC6*	*MAX*	*PMS2*	*UNG*
ATAD3C	***ESR1***	*MC1R*	*PNKP*	*UPK2*
ATG2A	*EXT1*	***MCL1***	*PPM1D*	*USP2*
ATM	*EYA4*	*MCPH1*	*PRDM2*	*USP7*
ATR	*FAM175A*	***MDM2***	*PRF1*	*VHL*
AXDND1	*FAN1*	*MDM4*	*PRKAR1A*	*WDR66*
AXIN2	*FANCA*	*MED12*	*PSD3*	*WNK1*
BABAM1	*FANCB*	*MEN1*	*PTCH1*	*WRN*
BAP1	*FANCC*	***MET***	***PTEN***	*WT1*
BARD1	*FANCD2*	*MFSD9*	*PTPN11*	*XPA*
BCCIP	*FANCE*	***MLH1***	*PYGB*	*XPC*
BLM	*FANCF*	*MLH3*	*RAD18*	*XRCC1*
BMPR1A	*FANCG*	*MPG*	*RAD50*	*XRCC2*
BPIFC	*FANCI*	*MRE11A*	*RAD51*	*XRCC3*
BRAF	*FANCL*	*MSH2*	*RAD51B*	*XRCC5*
BRCC3	*FANCM*	*MSH3*	*RAD51C*	*ZKSCAN3*
BRE	***FBXW7***	*MSH4*	*RAD51D*	*ZNF135*

BRIP1	*FGFR1*	*MSH5*	*RAD54B*	*ZNF493*
BUB1B	***FGFR2***	*MSH6*	*RASSF7*	*ZNF573*
C14ORF37	***FGFR3***	*MUTYH*	***RB1***	*ZNF695*
C1ORF86	***FGFR4***	*NBN*	*RBL1*	*ZNHIT1*
CAMKK1	*FH*	***NCOR1***	*RECQL*	*ZYG11A*
CASP8	*FIG4*	*NEIL1*	*RECQL4*	
CBFB	*FKBP7*	*NEIL2*	*RET*	
CCDC60	*FLCN*	*NEIL3*	*RINT1*	
CCND1	***FOXA1***	*NF1*	***RUNX1***	
CCND2	*FOXP1*	***NF2***	*RXFP4*	
CDC73	*GALNT15*	***NOTCH1***	*SALL2*	
CDCA7	***GATA3***	***NOTCH2***	*SDHA*	
CDH1	*GEN1*	***NOTCH4***	*SDHB*	
CDK4	*GJB6*	*NPC1*	*SDHC*	
CDK9	*GPR35*	*NPM1*	*SDHD*	
CDKN2A	*HAL*	*NPSR1*	***SF3B1***	
CENPF	*HIST1H2AH*	*NRAS*	*SLX4*	
CEP192	*HMGXB4*	*NRIP1*	***SMO***	
CHEK2	*HOXD9*	*NTHL1*	*SMUG1*	
CHR17:29230510-29230530	*HRAS*	*OGG1*	*SPEN*	
CHR17:29230520-29230522	*HSPBP1*	*OPRK1*	*ST20*	
CLK1	*IFNB1*	*ORC3*	***STK11***	
CTH	***IGF1R***	*OSBPL1A*	*STRADA*	
CTNNB1	*IMPDH1*	*PARP1*	*STYK1*	
DCLRE1A	***INPP4B***	*PARP2*	***TBX3***	
	KATNA1	*PARP3*	*TET2*	

Bold indicates genes of particular relevance to breast cancer. This panel was taken from previously published studies (1, 2).

Supplementary Table 2. Mutational landscape of pure, clonal and non-clonal papilloma

Gene	Genomic alteration	Transcript alteration	Protein alteration	Sample ID	Variant read proportion	Method/Validated
**Pure papilloma cases**						
PIK3CA	Chr3:178952085A>G	c.3140A>G	p.His1047Arg	P9	0.44	TSP/ND
ERBB3	Chr12:56482341G>T	c.889G>T	p.Asp297Tyr	P9	0.14	TSP/Yes
PIK3CA	Chr3:178936091G>A	c.1633G>A	p.Glu545Lys	P1	0.47	TSP/ND
HRAS	Chr11:533875G>T	c.181C>A	p.Gln61Lys	P1	0.13	TSP/ND
PIK3R3	Chr1:46511703CCAGGTTTTCTCATCATAATGGGG>C	c.1051_1073delCCCCATTATGATGAGAAAACCTG	p.Pro351ValfsTer3	P4	0.01	TSP/ND
TP53	Chr17:7576511CATTTTCAACTTACAAT>C	c.*98_*100+13delATTGTAAGTTGAAAAT	Splice site	P4	0.03	TSP/ND
PIK3CA	Chr3:178952085A>G	c.3140A>G	p.His1047Arg	P4	0.17	TSP/ND
SPEN	Chr1:16263691C>T	c.10060C>T	p.Pro3354Ser	P4	0.52	TSP/ND
PIK3CA	Chr3:178952085A>G	c.3140A>G	p.His1047Arg	P13	0.34	TSP/ND
PIK3CA	Chr3:178952085A>T	c.3140A>T	p.His1047Leu	P17	0.17	TSP/ND
PIK3CA	Chr3:178952085A>G	c.3140A>G	p.His1047Arg	P19	0.45	TSP/ND
PIK3CA	Chr3:178952085A>G	c.3140A>G	p.His1047Arg	P7	0.34	TSP/ND
PIK3CA	Chr3:178936091G>A	c.1633G>A	p.Glu545Lys	P21	0.39	TSP/ND
PIK3CA	Chr3:178952085A>G	c.3140A>G	p.His1047Arg	P18	N/A	Sanger
**Clonal cases**						
AKT1	Chr14:105246445A>C	c.155T>G	p.Leu52Arg	S5-pap	0.25	TSP/ND

AKT1	Chr14:105246445A>C	c.155T>G	p.Leu52Arg	S5-LG DCIS	0.37	TSP/ND
GATA3	Chr10:8115710AC>A	c.1060delC	p.Leu355Ter	S7-pap	0.15	TSP/ND
GATA3	Chr10:8115710AC>A	c.1060delC	p.Leu355Ter	S7-LG DCIS	0.21	TSP/ND
CBFB	Chr16:67063717G>T	c.165+1G>T	Splice site	S7-pap	0.28	TSP/Yes
CBFB	Chr16:67063717G>T	c.165+1G>T	Splice site	S7-LG DCIS	0.48	TSP/Yes
GATA3	Chr10:8115880T>TC	c.1229_1230insC	p.Ser411GlnfsTer97	S11-pap	0.24	TSP/ND
GATA3	Chr10:8115880T>TC	c.1229_1230insC	p.Ser411GlnfsTer97	S11-IG papillary DCIS	0.37	TSP/ND
ARID1A	Chr1:27093056A>AG	c.2987_2988insG	p.Lys997GlufsTer10	S16-pap	0.7	TSP/ND
ARID1A	Chr1:27093056A>AG	c.2987_2988insG	p.Lys997GlufsTer10	S16-HG DCIS	0.7	TSP/ND
SPEN	Chr1:16256280T>TG	c.3545_3546insG	p.Glu1183GlyfsTer8	S16-pap	0.66	TSP/ND
SPEN	Chr1:16256280T>TG	c.3545_3546insG	p.Glu1183GlyfsTer8	S16-HGDCIS	0.37	TSP/ND
PIK3R1	Chr5:67591104TTAA>T	c.1698_1700delTAA	p.Lys567del K567del	S16-pap	0.08	TSP/ND
PIK3R1	Chr5:67591107 AACCAGACCTTAT>A	c.1701_1712delACCAGA CCTTAT	p.Lys567_Ile571delinsA sn	S9-pap	0.33	TSP/ND
PIK3R1	Chr5:67591107 AACCAGACCTTAT>A	c.1701_1712delACCAGA CCTTAT	p.Lys567_Ile571delinsA sn	S9-G2IDC	0.42	TSP/ND
ARID1A	Chr1:27023501C>CA	c.607_608insA	p.His203GlnfsTer197	S1-pap	0.32	TSP/Yes
ARID1A	Chr1:27023501C>CA	c.607_608insA	p.His203GlnfsTer197	S1-LG DCIS	0.34	TSP/Yes
PIK3CA	WT	WT	WT	S6 (pap &	N/A	Sanger

				DCIS)		
**Non-clonal papilloma cases**						
PIK3CA	Chr3:178952085A>G	c.3140A>G	p.His1047Arg	S19-pap	0.3	TSP/ND
PIK3CA	Chr3:178936091G>A	c.1633G>A	p.Glu545Lys	S19-HG DCIS	0.16	TSP/ND
AKT1	Chr14:105246551C>T	c.49G>A	p.Glu17Lys	S12-pap	0.12	TSP/Yes
PIK3CA	Chr3:178952085A>G	c.3140A>G	p.His1047Arg	S18-pap	N/A	Sanger

ND: not done due to insufficient DNA; TSP: Targeted Sequencing Panel

Supplementary Table 3. Clonality Index (CI and CI2) of synchronous papilloma based on mutations

**Sample ID**	**CI (CI>0.8 = Clonal)**	**C12 (≥2.83 = Clonal)**
S1	1	6.13
S5	0.99	4.94
S7	1	28.78
S9	1	6.13
S11	1	6.13
S16	1	32.83
S19	0	1.94
S13	N/A (no shared mutations)	N/A (no shared mutations)

N/A: Not applicable

Supplementary Table 4. Genetic features absent in pure papilloma, but present in LG DCIS, HG DCIS, papillary carcinoma and clonal papilloma

	Number of cases (%)				Number of cases (%)	P value
**CNA associated with potential progression from papilloma to carcinoma**	Pure papilloma (n=24)	LG DCIS (n=21)#	HG DCIS (n=38)#	G1 PC (n=13)*	Clonal papilloma (n=11) (grades of carcinoma)	**Pure *vs* Clonal papilloma**
1q gain	0	10/21 (47.6%)	19/38 (50%)	11/13 (85%)*	6/11 (55%) (4 LG, 2 IG)	**0.0002**
11q loss	0	5/21 (23.8%)	15/38 (39.5%)	N/A	5/11 (45%) (1 LG, 2 IG, 2 HG)	**0.001**
11q13.1-q13.4 high gain	0	4/21 (19%)	10/38 (26.3%)	N/A	2/11 (18%) (2 IG)	0.09
13q loss	0	1/21 (4.8%)	13/38 (34.2%)	N/A	2/11 (18%) (1 LG, 1 HG)	0.09
13q gain	0	0	5/38 (13.2%)	N/A	1/11 (9%) (HG)	0.3
3p loss (partial)	0	0	7/38 (18.5%)	N/A	1/11 (9%) (1 LG)	0.3
3q gain (3q26.1-29)	0	0	6/38 (16%)	2/13 (15%)*	2/11 (18%) (1 LG, 1 HG)	0.09
Both 8p loss & 8q gain	0	0	14/38 (37%)	N/A	1/11 (9%) (HG)	0.3
20q gain	0	WC 20 1/21 (4.8%); 20q13.2 (7 Mb) 1/21;<br>Total 2/21 (9.5%)	19/38 (50%)	N/A	2/11 (18%) (1IG, 1HG)	0.09
6q loss	0	3/21 (14.3%) (2/21 partial loss,1/21 full arm loss)	6/38 (15.8%) (partial loss n=5)	N/A	1/11 (9%) (1 LG)	0.3
14q loss	0	0	13/38(34%)	N/A	1/11 (9%) (1 HG)	0.3

22q loss	0	4/21 (19%)	8/38 (21.1%)	N/A	1/11 (9%) (1HG)	0.3
10p gain	0	0	6/38 (15.8%)	N/A	1/11 (9%) (IG)	0.3
WC 7 gain	0	0	7/38(18.4%)	2/13 (15%)*	1/11 (9%) (LG)	0.3
X gain	0	4/21 (19%)	3/38 (7.8%)	2/13 (15%)*	1/11 (9%) (HG)	0.3
18q gain	0	0	0	N/A	1/11(9%) (HG)	0.3

P value from Fisher Exact test (significant P values are in bold). CNA: copy number alterations; WC: Whole chromosome.# previously published studies (2-4) * Studies are done by FISH. N/A: data not Available. Data for G1 PC was taken from previous publications (5-7).

Supp Tables References:

1. Lee JEA, Li N, Rowley SM, Cheasley D, Zethoven M, McInerny S, et al. Molecular analysis of PALB2 associated breast cancers. The Journal of pathology. 2018.
2. Pang J-MB, Savas P, Fellowes AP, Arnau GM, Kader T, Vedururu R, et al. Breast ductal carcinoma in situ carry mutational driver events representative of invasive breast cancer. Modern Pathology. 2017;30:952-963.
3. Kader T, Hill P, Zethoven M, Goode DL, Elder K, Thio N, et al. Atypical ductal hyperplasia is a multipotent precursor of breast carcinoma. The Journal of Pathology. 2019;248(3):326-38.
4. Gorringe KL, Hunter SM, Pang JM, Opeskin K, Hill P, Rowley SM, et al. Copy number analysis of ductal carcinoma in situ with and without recurrence. Mod Pathol. 2015;28(9):1174-84.
5. Tsuda H, Takarabe T, Inazawa J, Hirohashi S. Detection of numerical alterations of chromosomes 3, 7, 17 and X in low-grade intracystic papillary tumors of the breast by multi-color fluorescencein situ hybridization. Breast Cancer. 1997;4(4):247-52.
6. Tsuda H, Takarabe T, Susumu N, Inazawa J, Okada S, Hirohashi S. Detection of numerical and structural alterations and fusion of chromosomes 16 and 1 in low-grade papillary breast carcinoma by fluorescence in situ hybridization. The American journal of pathology. 1997;151(4):1027.
7. Tsuda H, Uei Y, Fukutomi T, Hirohashi S. Different incidence of loss of heterozygosity on chromosome 16q between intraductal papilloma and intracystic papillary carcinoma of the breast. Cancer Science. 1994;85(10):992-6.

Supplementary File 1: Sample Information

Supp File 1a-Pure papilloma cases

Sample ID	Method utilised	CNA/mutation	Age at diagnosis (years)	Follow up (years)	Specimen	Benign/Atypical	Other Lesions	calcification	Symptoms	FGA	PIK3CA	ER	P63	CK5/6	Ki67(%)
P1	TSP	both	72	15	core	benign	.		lump	0%	Y	+	+	+	2%
P2#	LC WGS	CN, PIK3CA(sanger)	68	13	core	benign	no	Y		0%	N	+	+	+	0
P3	MIP	CN	33	13	core	atypical papillary lesion	sclerosing adenosis			0%		+	+	+ve (small populations -ve)	0
P4	TSP	both	76	13	HNL	benign papilloma w UDH	no			0%	Y	+	+	+	1.6
P5#	LC WGS	CN, PIK3CA (sanger)	44	13	microdochectomy	benign	UDH, apocrine, sclerosing adenosis		ND	2%	N	+	+	+	0
P6#	LC WGS	CN	69	13	excision	sclerosed intraductal papilloma, no atypia	no		lump	0%		+	+	+	1.5
P7	TSP	both	60	13	lumpectomy	atypical papillary lesion	no		lump	5%	Y	+	+	+ (few -ve)	1.3
P8	LC WGS	CN	52	12	core	atypical papillary leison	.		lump	5%		+	+	+ (small populations -ve)	2.1
P9*	TSP	both	63	10	core	benign	.			0%	Y	+	+	+	0
P10	MIP	CN	76	11	core	benign	no			0%		+	+	+	N/A
P11	MIP	CN	51	10	core	benign	no	Y		0%		+	+	+	N/A
P12	MIP	CN	75	10	core	benign	no			0%		+	+	+	2
P13*	TSP	both	20	9	excision	benign	.			2%	Y	+	+	+	0.6
P14	LC WGS	CN	57	9	core	benign	.	Y		1%		+	+	+	3
P15#	LC WGS	CN, PIK3CA (sanger)	64	9	core	benign				0%	N	+	+	+	3.2
P16	MIP	CN	60	7	core	benign	no	Y		0%		+	+	+	1.5
P17	TSP	both	50	7	excision	benign	.		ND	15%	Y	+	+	+	4
P18	LC WGS	CN, PIK3CA (sanger)	74	7	excision	benign	ADH			4%	Y	+	+	+	5.4
P19	TSP	both	62	6	core	benign	.			3%	Y	+	+	+	10%
P20	MIP	CN	57	6	core	benign papilloma w UDH	ADH, apocrine metaplasia	Y		0%		+	+	+	1.5
P21	TSP	both	50	5	excision	benign	.			0%	Y	+	+	+	N/A
P22	MIP	CN	58	5	core	benign papilloma w UDH	no			1%		+	+	+	1
P23	MIP	CN	48	21	core	atypical papillary leison	.	Y		0%		+	+	+ (small populations	N/A

														-ve)		Y=Ye
P24	TSP	both	68	20	core	benign	.		lump	0%	N	+	+	+	0%	s;

N/A: Not available; CN: Copy number; * noisy CN profile; # normalised against normal to reduce noise and spurious CN calls; ND=nipple discharge; LC WGS: Low-coverage Whole Genome Sequencing, MIP: Molecular Inversion Probe SNP arrays, TSP: Targeted Gene Panel Sequencing; UDH: usual ductal hyperplasia, ADH: atypical ductal hyperplasia.

Supp File 1b-synchronous papilloma cases

**Low grade Cancer**																
**Sample ID**	**Lesions**	**Technique used**	**CN/mutation**	**Clonal**	**ER/PR/Her2 (cancer)**	**Specimen**	**laterality**	**tumour arch feature**	**Block**	**Age (years)**	**Extra comment**	**Other lesion/symptoms**	**ER**	**P63**	**CK5/6**	**Ki67**
S1	benign pap, LG DCIS	TSP	both	Yes	ER+, Her2-(Copy number/CN)	WLE	L	6mm dimension LG DCIS	same	48		lump	N/A	+	+	pap 2.5%, DCIS 4.7%
S2	atypical pap,LG DCIS	LCWGS	CN	Yes	ER+, Her2-(Copy number/CN)	excision	L	5 mm dimension	diff	83		ADH, UDH	+	+	+ (small -ve populations)	pap 1.3%, DCIS 11%
S3	atypical papillary lesion, LG DCIS	LCWGS	CN	yes	ER+, Her2-(Copy number/CN)	WLE	R	cribriform LG DCIS	same	67		ADH	+	+	+ (small -ve populations)	N/A
S4	Papillomatosis benign, LG DCIS	MIP	CN	No	N/A	WLE	R	N/A	same	41		N/A	N/A	N/A	N/A	N/A
S5	atypical papillary leison,LG DCIS	TSP	both	Yes	ER+PR+Her2-	stereo core	R	5 mm, calcified	diff	40		lump, CC	+	+	+ (small -ve populations)	pap 5.8%, DCIS 6%
S6	atypical papillary lesion , LG DCIS	LCWGS	CN, PIK3CA (sanger)	Yes	ER+Her2-	WLE	N/A	6 mm dimension	same	72		N/A	+	+	+ (small -ve populations)	N/A
S7	atypical papillary lesion, LG-DCIS	TSP	both	Yes	ER+PR+ Her2-	core	R	cribriform	same	50		N/A	+	+	+ (small -ve populations)	3% pap, 6.1% DCIS
**Intermediate grade Cancer**																
**Sample ID**	**Lesions**	**Technique used**	**CN/mutation**	**Clonal**	**ER/PR/Her2 (cancer)**	**Specimen**	**laterality**	**tumour arch feature**	**Block**	**Age (years)**		**Other lesion/symptoms**	**ER**	**P63**	**CK5/6**	**Ki67**
S8	papillomatosis. G2 IDC	LCWGS	CN, PIK3CA (sanger)	No	ER+ PR+ Her2 equivocal(path) Her2-(CN)	partial mastectomy	L	1-2 mm; solid and cribriform, no comedo-necrosis	same	70		symp N/A, not niple discharge	+	+	+	pap 4.9%, IDC 2.1%
S9	atypia papillary lesion, G2 IDC	MIP & TSP	both	Yes	ER+PR+ Her2-	lump excision	R	12 mm	same	59		lump	+	+(path)	+ (small -ve populations)	pap 1.6%, IDC 7.3%
S10	atypia papillary lesion, IG DCIS	LCWGS	CN	Yes	N/A	WLE	L	N/A	same	59		N/A	N/A	+ (path)	N/A	N/A
S11	atypia pap, papillary IG DCIS, G1 mucinous	TSP, LCWGS(mucinous)	both	Yes	ER+PR+; SISH not amplified (only mucinous reported)	HN excision	L	25 mm mucinous, solid/cribriform	same	45	Her 2+ pap, DCIS (amplified 17q12-q21.2) not mucinous	CC w atypia	+	N/A poor staining	N/A poor staining	4.7% DCIS, pap 2% (poor stain), 30% mucinous
S12	IG DCIS,beingn pap	LCWGS(DCIS), pap(TSP)_sanger (AKT1)	both	No	N/A	WLE	R	25 mm dimension solid & cribriform w comedo-necrosis	diff	71		no pagets disease	+	+	+ve	N/A
S13	atypia pap,IG-DCIS	TSP	both	No	ER+PR+Her2-	WLE	L	cribriform	diff	69		UDH, CC	+	N/A poor staining	N/A poor staining	N/A

High grade Cancer																
**Sample ID**	**Lesions**	**Technique used**	**CN/mutation**	**Clonal**	**ER/PR/Her2 (cancer)**	**Specimen**	**laterality**	**tumour arch feature**	**Block**	**Age (years)**		**Other lesion/symptoms**	**ER**	**P63**	**CK5/6**	**Ki67**
S14	Papillomatosis benign, HG DCIS, G2 IDC	LCWGS	CN	No	ER+PR+ Her2+(CN)	WLE	L	DCIS-cribriform & comedo, necrosis	diff	71		UDH, sclerosing adenosis	N/A	N/A	N/A	N/A
S15	benign pap, LG_IGDCIS,G3IDC	LCWGS	CN, PIK3CA (sanger)	No	ER+PR+Her2-	WLE	L	8mm IDC	diff	57		N/A	+	+	+	pap 0%, DCIS 4.1%
S16	atypical papillary lesion, HG DCIS	MIP, TSP	both	Yes	ER+PR-Her2-	excision	R	25 mm dimension of DCIS	same	66		N/A	+	+	+ (small -ve populations)poor staining	pap 9.6%, DCIS 12%
S17	benign papilloma, HGDCIS	LCWGS	CN	No	ER+Her2-	WLE	N/A	40 mm comedo necrosis	same	49		N/A	+	N/A	+	24.2%DCIS, Pap-0%
S18	atypia pap, IG-HG DCIS	LCWGS	CN, PIK3CA (sanger)	No	ER+PR+Her2- (CN)	WLE	R	solid	same	58		N/A	+	+	+ (small -ve populations)	12.5% pap, 15.5% DCIS
S19	benign pap,IG-HG DCIS	TSP	both	No	Her2+(CN)	partial mastectomy	L	clinging	diff	63		paget's disease	+	+	+	1.2% pap, 14.5% DCIS
S20	atypical pap, HG DCIS	MIP	CN	Yes	Her2-(CN),ER+	excision	N/A	N/A	same	N/A		N/A	N/A	N/A	N/A	N/A

N/A: Not available; L: Left, R: Right; CN: Copy Number; LG: Low grade, HG: High grade, IG: Intermediate grade, G1: Grade 1, G2: Grade 2, G3: Grade 3; WLE: wide local excision

Supplementary File 2- DNA input, sequencing performance

sample ID	Block	Lesion type	Sequencing method	VBPCR (bp)	Library final input(ng)	Coverage (x)	Fragment length (bp)	Total reads (millions)
P9	1A	Pure benign pap	TSP	300	100			
P13	1E	Pure benign pap	TSP	300	38.8			
P14	1A	Pure benign pap,calc	LCWGS	300	20	0.89	157	39.10
P15	1A	Pure benign	LCWGS	300	13	1.37	113	61.10
P17	1B	Pure benign pap	TSP	300	65.7			
P19	1A	Pure benign pap	TSP	300	38.4			
P21	1F	Pure pap	TSP	300	150			
S18	3I	synchronous-pap	LCWGS	300	20	1.86	162	81.21
S18	3I	synchronous-DCIS	LCWGS	300	20	1.62	147	70.76
S11	1B	synchronous-atypia pap	TSP	300	100			
S11	1B	synchronous-DCIS	TSP	300	100			
S11	1B	synchronous-mucinous	LCWGS	300	20	1.16	138	50.31
S13	1P	synchronous-atypia pap	TSP	300	100			
S13	1I	synchronous-DCIS	TSP	300	150			
S19	1D	synchronous-pap	TSP	400	100			
S19	1C	synchronous-DCIS	TSP	400	100			
S2	1B	synchronous-atypical pap	LCWGS	200	20	1.32	74	68.20
S2	1I	synchronous-DCIS	LCWGS	100	9	0.97	73	48.59
S8	1C	synchronous-pap	LCWGS	200	20	1.65	87	78.03
S8	1C	synchronous-IDC	LCWGS	200	20	1.38	99	61.91
S5	1A	synchronous-pap	TSP	300	100			
S5	2A	synchronous-DCIS	TSP	200	100			
S12	3G	synchronous-pap	TSP	300	150			
S12	3E	synchronous-DCIS	LCWGS	300	20	1.21	144	52.78
S7	1A	synchronous-pap	TSP	300	103.2			
S7	1A	synchronous-DCIS	TSP	300	150			
P18	1I	Pure benign pap	LCWGS	200	20	1.81	124	80.24
S1	1E	synchronous-Pap	TSP	200	217			
S1	1E	synchronous-DCIS	TSP	200	318			
S15	1C	synchronous-Pap	LCWGS	200	25	1.58	105	70.34
S15	1A	synchronous-DCIS	LCWGS	200	25	1.46	98	65.85
S15	1A	synchronous-IDC	LCWGS	200	20	1.37	86	63.66
S3	D	synchronous-pap	LC WGS	N/A	10	1.52	126	68.22
S3	D	synchronous-DCIS	LC WGS	N/A	8	1.32	105	61.00
S10	C	synchronous-pap	LC WGS	N/A	10	1.27	142	55.15

S10	C	synchronous-DCIS	LC WGS	N/A	20	1.13	162	49.27
P2	B	Pure pap	LCWGS	N/A	5	1.8	130	82.25
P5	C	Pure pap	LCWGS	N/A	5	1.45	118	68.91
P6	D	Pure pap	LCWGS	N/A	5	1.12	109	70.58
P8		Pure pap	LCWGS	N/A	5	0.75	79	40.26
S6	1A	synch-pap	LCWGS	300	5	1.07	138	46.60
S6	1A	synch-DCIS	LCWGS	300	20	0.95	128	42.06
S17	1H	synch pap	LCWGS	600	5	1.14	144	49.61
S17	1H	synch-DCIS	LC WGS	600	5	2.1	161	92.96

LC WGS: Low-coverage Whole Genome Sequencing; TSP: Targeted Gene Panel Sequencing ; VBPCR: van Beers PCR (quality of DNA).
